# CyberDetect MLP a big data enabled optimized deep learning framework for scalable cyberattack detection in IoT environments

**DOI:** 10.1038/s41598-025-24459-w

**Published:** 2025-11-19

**Authors:** Talluri Upender, M. Neelakantappa, C. Prakasa Rao, Jaideep Gera, Vuyyuru Lakshma Reddy, Nagendar Yamsani

**Affiliations:** 1 Department of Computer Science and Engineering, CMR College of Engineering & Technology, Hyderabad, Telangana India; 2 Department of IT, Vasavi College of Engineering, Hyderabad, Telangana India; 3 Department of CSE, Bapatla Engineering College, Bapatla, Andhra Pradesh India; 4 Department of Computer Science & Business Systems (CSBS), RVR & JC College of Engineering, Guntur, Andhra Pradesh India; 5 Department of Computer Science & Engineering, Koneru Lakshmaiah Education Foundation, Green Fileds, Vaddeswaram, Guntur, 522302 AP India; 6 School of Computer Science and Artificial Intelligence, SR University, Warangal, India

**Keywords:** IoT security, Cyberattack detection, Big data analytics, Deep learning, Intrusion detection system, Computer science, Information technology, Scientific data, Environmental sciences, Environmental social sciences

## Abstract

The rapid growth in the adoption of Internet of Things (IoT) ecosystems has led to a large-scale influx of multidimensional data, highlighting vast attack surfaces that diverse types of cyber threats can exploit. However, existing traditional intrusion detection systems (IDS) and many common machine learning (ML) models do not scale very well. They are unfortunately not interpretable and unable to deal with high-dimensional significant data streams, which makes them very limited for use in large-scale IoT applications. In this paper, we propose CyberDetect-MLP, a scalable, explainable, big data-enabled, and optimized deep learning framework for IoT cyberattack detection, addressing these challenges. We present a robust framework that employs Apache Spark for distributed ingestion and preprocessing, Mutual information–based feature selection, and a multi-layer perceptron (MLP) with batch normalization, dropout, and cosine annealing scheduling to improve performance and generalization. To enhance transparency and ensure trust from the administrator, an optional explainable AI (XAI) module is added utilizing Grad-CAM and SHAP. Extensive experiments on the full TON_IoT dataset show that CyberDetect-MLP outperforms the baselines of Random Forest, XGBoost, and vanilla MLP with an accuracy of 98.87% and a ROC-AUC of 99.10%. Ablation studies and explainability evaluations further corroborate the framework’s robustness and the trustworthiness of the results. In contrast to existing methodologies, the proposed paradigm closes the gap between big data analytics and interpretable deep learning in cybersecurity to provide an end-to-end IDS approach specifically targeting real-time smart city, industrial IoT, and critical infrastructure applications. To ensure reproducibility and transparency, the complete implementation of the proposed CyberDetect-MLP framework, including data preprocessing, model training, and evaluation scripts, is publicly available at https://github.com/upender0123/CyberDetect-MLP.

## Introduction

The exponential growth of Internet of Things (IoT) devices across various sectors, including smart cities, industrial automation, and healthcare, has resulted in the generation of massive volumes of real-time, heterogeneous data. While this evolution enhances connectivity and automation, it also expands the attack surface for cyber threats targeting resource-constrained IoT endpoints and communication protocols. Traditional intrusion detection systems (IDS), primarily based on signature or rule-based mechanisms, have become insufficient in detecting novel or evolving threats within such dynamic environments. Consequently, data-driven and learning-based security analytics have gained traction for their ability to detect complex and previously unseen patterns.

Recent literature has explored the application of machine learning (ML) and deep learning (DL) models to intrusion detection^1,2^, with notable success in static and structured environments. However, many existing models exhibit limitations in scalability, interpretability, and adaptability to the large-scale IoT deployments that generate big data^3,4^. Moreover, classical ML models struggle with high-dimensional data, while deep architectures, though powerful, often operate as black boxes. A lack of integrated frameworks remains that combine significant data infrastructure, optimized deep learning, and explainable AI to deliver a robust and interpretable solution for IoT cyberattack detection.

While in some domains, this feature is effective for identifying malicious IoT traffic, the mentioned limitations indicate the importance of having a one-stop IDS for analyzing high-volume heterogeneous IoT traffic while providing acceptable scalability and interpretability. Flexibility to adapt to dynamic threats, real-time big data analytics, and transparency through reproducible decision-making for system administrations are the key requirements of Real-World IoT ecosystems.

To address these challenges, this research proposes CyberDetect-MLP, a Big Data-enabled Optimized Deep Learning Framework designed for scalable cyberattack detection in IoT environments. The primary objective is to develop an accurate, scalable, and explainable intrusion detection system (IDS) utilizing distributed data processing (Apache Spark), a custom-tuned multi-layer perceptron (MLP) architecture, and Grad-CAM-based interpretability. Key novelties include the integration of distributed preprocessing via Spark MLlib, optimized MLP configuration tailored for high-dimensional IoT data, and the incorporation of heatmap-based explainability to enhance model transparency.

In contrast to regular big data, IoT Big Data is multi-source and highly heterogeneous telemetry (sensors, devices, logs), high-velocity streaming with concept drift over time, and also with strict edge/fog processing constraints and even stronger privacy/security demands. They necessitate an architecture that can support distributed real-time analytics, adaptively learn as traffic patterns change over time, and explain the prediction to be compliant with regulations and establish trust.

- Although many IDS frameworks for IoT security have been presented in previous works so far, most of them either use standard ML/DL models while lacking essential interpretability features necessary for real deployment, or disregard the scalability of big data use for building IoT applications. Unlike the current work, we present an integrated big data pipeline (Kafka/Flume–HDFS–Spark) along with an optimized multi-layer perceptron (MLP) architecture, which is tailored to high-dimensional IoT telemetry. Moreover, Mutual Information–based feature selection is applied to minimize computational costs, whilst explainable AI (Grad-CAM and SHAP) is used to enhance transparency in predicted explanations. Our work is unique due to this simultaneous consideration of scalability, interpretability, and systematic assessment on the complete TON_IoT dataset. The main contributions of this work can be summarized as follows:
- CyberDetect-MLP Framework Development: A big data–enabled intrusion detection framework comprising a seamless integration of Apache Kafka/Flume for scalable data ingestion, Hadoop HDFS for distributed storage, and Apache Spark for parallel preprocessing and feature engineering.
- Fine-grained Deep Learning Model: A custom 8-layer perceptron (MLP) with batch normalization, dropout regularization, and a cosin-annealing learning rate scheduler, tailor-made for high-dimensional IoT telemetry data.
- Feature Selection for Efficiency. Mutual Information-based feature selection is used to select the relevant features and to reduce the dimension of the data, where the vital attributes are preserved, which helps to enhance the performance and efficiency of training.
- Explainable AI Integration: To instill trust in model predictions by administrators, we introduce Grad-CAM and SHAP–based interpretability modules on top of it, such that one can have a transparent overview of why the model predicted this.
- Extensive Empirical Validation: We perform extensive experiments based on the TON_IoT dataset, obtaining an accuracy of 98.87%, outperforming multiple baseline models (i.e., Random Forest, XGBoost, vanilla MLP) with extensive ablation studies and explainability analyses.

The remainder of this paper is structured as follows: Sect. 2 reviews the relevant literature across machine learning (ML), deep learning (DL), and big data frameworks for intrusion detection systems (IDS). Section 3 outlines the proposed methodology, which includes system design, dataset preparation, model implementation, and deployment strategy. Section 4 presents experimental results, comparisons, and visualization-based analyses. Section 5 discusses key insights and limitations of the study. Finally, Sect. 6 concludes the paper and outlines directions for future work.

## Related work

This section reviews existing approaches in cyberattack detection for IoT environments, focusing on traditional machine learning models, deep learning architectures, and big data-enabled intrusion detection frameworks. The review identifies performance limitations, lack of scalability, and poor interpretability in current methods, thereby establishing the research gap that motivates the development of the proposed CyberDetect-MLP framework.

### Machine learning and deep learning approaches for cyberattack detection

Extensive research has explored classical machine learning (ML) and advanced deep learning (DL) techniques for detecting cyberattacks across diverse networks. These methods enhance intrusion detection by learning patterns from labeled traffic data using algorithms like SVM, Random Forest, k-NN, CNNs, RNNs, LSTM, and hybrid models. Krishnashree Achuthan et al.^1^ examined the integration of cybersecurity and environmental sustainability, highlighting important topics such as the application of blockchain in smart grids and the use of artificial intelligence in threat detection. The literature’s limited reach is one of its limitations; nevertheless, future research indicates more comprehensive SDG comparisons.

Abid Mohamed Nadhir et al.^2^ present a Reinforcement Learning-based cybersecurity strategy for healthcare IoT utilizing PPO and A2C models in this research. PPO received fewer prizes than A2C. One of CyberBattleSim’s limitations is its inability to replicate the intricacies of the real world. Improvements to real-world modeling were proposed for future development. Tin Lai et al.^3^ present an ensemble machine learning method for IoT cybersecurity, which utilizes Bayesian optimization to detect anomalies. It demonstrates the effectiveness of tree-based models, such as XGB and GBM. In the future, various protocols and privacy-preserving strategies will be investigated.

Sankaramoorthy Muthubalaji et al.^4^ introduce the AEFS-KENN big data AI system for intelligent grid intrusion detection, which achieves 99.5% accuracy, in this paper. While efficient, its security reach is constrained; cryptographic integration is a future development. Noha Hussen et al.^5^ introduced FSBDL, a real-time intrusion detection system that achieves 99.93% accuracy by utilizing an optimized CNN with the Adam and RMSprop optimization algorithms. It is effective but has limited explainability; interpretability will be investigated in a subsequent study.

Faheem Ahmad et al.^6^ examined the dual function of big data in cybersecurity, emphasizing both machine learning and encryption, as both a target and a tool. It takes note of present issues and trends and projects future R&D expansion. Vinden Wylde et al.^7^ The report focuses on data privacy and legal concerns, highlighting the challenges in implementing blockchain technology for cybersecurity. The framework it suggests combines visualization, machine learning, and big data to improve security. Enhancing transparency and confidence in digital systems is the framework’s primary goal. SMEs face difficulties in adopting blockchain in real time due to its network latency. To encourage wider usage, further work will solve these latency difficulties and improve legal frameworks.

George Bravos et al.^8^ presented a CNN-LSTM hybrid model for IoT intrusion detection, which achieved an accuracy of 99.52% on the CICIDS2017 dataset. Although it excels at real-time detection, it needs to be enhanced for more comprehensive protection against diverse and sophisticated attacks. Iqbal H. Sarker^9^ offered a CNN-LSTM hybrid model that achieves 99.52% accuracy on CICIDS2017 for real-time IoT intrusion detection. Although it has high accuracy, it requires more testing and a substantial amount of training data. Mohamedamineferrag et al.^10^ compared RNN, CNN, and DNN on actual datasets to examine federated deep learning for IoT cybersecurity. It highlights vulnerabilities, demonstrates increased accuracy and privacy, and makes recommendations for future security improvements.

Mahmoudelsisi et al.^11^ propose a unique CPS-based IoT architecture for precise defect and cyberattack detection, featuring clear visualization and utilizing XGBoost for real-time GIS monitoring. Future work will encompass larger power system applications. Mrutyunjaya Panda et al.^12^ proposed an accurate, low-complexity machine learning and deep learning method for identifying IoT botnets. Future research will combine blockchain technology with edge and cloud computing to enable real-time detection and monitoring. Nighat Usman et al.^13^ presented a hybrid strategy for predicting IP reputation that combines data forensics, machine learning, and dynamic malware analysis. It enhances detection, reduces false alarms, and recommends future integration of firewall and antivirus systems. Aymen Yahyaoui et al.^14^ described a real-time intrusion detection system for IoT networks that uses Spark Streaming and Apache Flink to achieve excellent accuracy and speed. Other engines and anomaly detection kinds will be investigated in future development. Shahid Latif et al.^15^ presented DnRaNN, which achieves 99.14% accuracy in binary and 99.05% accuracy in multiclass situations for IoT intrusion detection. In the future, FPGA integration will be used to improve hardware performance.

Jingyu Liu et al.^16^ suggested PSO-LightGBM for IoT intrusion detection, which increases detection rates for assaults with small sample sizes (such as shellcode and backdoor). Future research will incorporate multi-task learning to improve performance. Nivedita Mishra and Sharnil Pandya^17^ examined IoT security with an emphasis on DDoS attacks, intrusion detection systems, and machine learning and deep learning-based mitigation strategies. The goal of future research is to create a reliable, all-purpose intrusion detection system. João Vitorino et al.^18^ use the IoT-23 dataset in the study to examine supervised, unsupervised, and reinforcement learning approaches for IoT intrusion detection. The best-performing model was LightGBM, and future research will combine models to improve detection.

Mohanad Sarhan et al.^19^ utilize three datasets in their evaluation of the NetFlow and CICFlowMeter feature sets for machine learning-based intrusion detection. NetFlow demonstrated better detection precision. The model’s explainability was assessed using the SHAP method. More extensive evaluations will be part of future work. Joseph Bamidele Awotunde and Sanjay Misra et al.^20^ proposed a hybrid AI model that achieves a 99.75% detection rate and 99.45% accuracy for IoT intrusion detection. It outperforms current models and provides recommendations for future advancements that utilize genetic algorithms for feature selection.

Souradip Roy et al.^21^ presented a lightweight machine learning (ML)-based intrusion detection model (B-Stacking) that is tailored for the Internet of Things. It has been evaluated on CICIDS2017 and NSL-KDD, demonstrating high accuracy, minimal false alarms, and outperforming other models. Adeel Abbas et al.^22^ presented an ensemble intrusion detection system (IDS) that uses logistic regression, naive Bayes, and decision trees with hard voting. It has been evaluated on CICIDS2017 and has demonstrated good accuracy, a low false alarm rate, and resource efficiency. Although these ML/DL methods provide superior detection rates, most are evaluated on small/old datasets and omit necessary components for classifying high-velocity IoT traffic or provisioning interpretability. In this work, we address these challenges by integrating a big data pipeline with an explainable, optimized MLP architecture.

### IoT-specific intrusion detection techniques

With the proliferation of IoT devices, specialized IDS solutions have emerged to handle resource constraints, protocol diversity, and limited training data. These include lightweight models, flow-based detection, federated learning, and anomaly detection optimized for edge computing. Vinayakumar Ravi et al.^23^ introduced a GRU-based deep learning intrusion detection system for SDN-IoT that uses kernel-PCA and feature fusion to achieve improved accuracy and generalizability. However, it requires adversarial robustness analysis and optimization. Ajay Kumar et al.^24^ introduced a network-based intrusion prevention system (NBIPS) for Internet of Things (IoT) security, which has been validated using signature-based detection. It provides robust protection but requires further development because it is not scalable or AI-based. YakubKayodeSaheed et al.^25^ proposed an ML-IDS for IoT that achieves 99.9% accuracy by utilizing feature scaling, principal component analysis (PCA), and supervised learning. Although it has scalability issues, it produces good results; later research looks at ensemble approaches.

Pampapathi B M et al.^26^ recommended a Filtered Deep Learning Model for IDS in IoT, which outperforms DLNN/ANN on the TON_IoT dataset with 96.12% accuracy. It utilizes MQTT/AQMP and LFEHO for data brokering; protocol and algorithm improvements are suggested for future work. P. L. S. Jayalaxmi et al.^27^ examined IDS/IPS models with ML/DL, presenting a new risk factor mapping framework that contrasts with current approaches, identifies advantages and disadvantages, and suggests future lines of inquiry for improved IoT security.

Muhammad Asif et al.^28^ introduced MR-IMID, a MapReduce-ML model that uses artificial neural networks (ANN) for real-time IoT intrusion detection. It has a 97.6% accuracy rate, is scalable, has fewer false alarms, and has room for improvement. Mouaad Mohy-Eddine et al.^29^ proposed a hybrid intrusion detection system (IDS) for the Industrial Internet of Things (IIoT) that utilizes RF with PCC and IF for feature selection and outlier elimination. It achieves up to 99.99% accuracy and plans to validate its performance in a broader dataset. Shiyu Wang et al.^30^ introduced Res-TranBiLSTM, a spatiotemporal deep learning intrusion detection system that utilizes ResNet, Transformer, and BiLSTM to achieve an accuracy of up to 99.56%. The goals of future work include unsupervised learning and real-world deployment.

Fuhua Huo^31^ presented an IoT-based cloud intrusion detection system that uses Mings distance and data clustering to achieve excellent accuracy with less than 0.3% of detections missed; further research could enhance real-time adaptability. Alireza Zohourian et al.^32^ introduce IoT-PRIDS, a lightweight, non-ML host-based intrusion detection system that utilizes packet representations in this study. It has achieved good CICIoT2023 results with few false positives; further research aims to enable dynamic device adaptation. Mohanad Sarhan et al.^33^ assessed PCA, AE, and LDA using six machine learning models on three NIDS datasets, demonstrating the necessity for a broad baseline feature set and finding that no single optimal combination exists.

Ajay Kaushik and Hamed Al-Raweshidy^34^ presented TLBO-IDS, an IoT-optimized low-overhead intrusion detection system that outperforms GA and bat methods by as much as 40% on UNSW-NB15; future research attempts to incorporate encryption standards. Amritpal Singh et al.^35^ introduced SecureFlow, a dual-layer IDS and dynamic rule configuration system for IoT based on Software-Defined Networking (SDN), which exhibits good emulation results. Future research will focus on the real-time deployment and broader validation of attacks.

Ibrahim A. Fares et al.^36^ presented the TFKAN Transformer for IoT IDS, utilizing Kolmogorov-Arnold Networks, which achieves up to 99.96% accuracy with 78% fewer parameters. Real-time application and training improvement are the goals of future work. Mahmoud Ragab et al.^37^ introduced NGCAD-EDLM, an ensemble CNN-DBN model for IIoT cybersecurity that utilizes HBA and LEOA, achieving 99.21% accuracy. Future work aims to enhance adaptability and reduce complexity.

While recent IoT-oriented IDS solutions have explored fog architectures, threat prediction using LLM, and early botnet detection, they operate in isolated settings that are not scalable and lack a combination of big data analytics with transparent model explanations. CyperDetect-MLP provides a bridging architecture with a single Spark-enabled (backend) architecture and XAI modules.

### Big data frameworks for security analytics

Big data technologies such as Hadoop and Spark have been adopted to process high-velocity security data. These frameworks support scalable data ingestion, transformation, and distributed model training, enabling real-time and batch analytics over massive IoT traffic. Rania Aboalela et al.^38^ presents HFPODL-DDoSCD, a deep learning-based IoT DDoS detection model with 99.52% accuracy, utilizing STO and APO optimizations. The goals of future research include real-time edge deployment and broader generalization. Olanrewaju-George et al.^39^ introduces FL-trained supervised and unsupervised DL models for IoT IDS, demonstrating that AE-FL performs best on the N-BaIoT dataset, offering privacy advantages but being constrained by device-specific tuning requirements. Hui Chen et al.^40^ To improve performance in dynamic contexts with class imbalance handling and quantization, this research suggests the SICNN model for IoT intrusion detection. It performs better than existing models when tested on the CIC-IDS 2017 and CICIoT 2023 datasets.

Vanlalruata Hnamte and Jamal Hussain^41^ proposed a hybrid DCNNBiLSTM-based intrusion detection model that achieves high accuracy by utilizing the Edge_IIoT and CICIDS2018 datasets. Although it takes longer to train than single models, it performs better; real-time deployment will be a part of future work. Mimouna Abdullah Alkhonain et al.^42^ presented the SPOHDL-ID model, which combines Blockchain, Sandpiper Optimizer, CNN-SAE, and BSOA for IoT intrusion detection. It has been tested on ToN-IoT and CICIDS-2017, achieving an accuracy of approximately 99.5%; however, scalability and resource requirements remain issues. C. Rajathi and P. Rukmani^43^ introduced a Hybrid Learning Model that combines parametric and non-parametric classifiers through stacking. It has been tested on NSL-KDD, UNSW-NB15, and CICIDS2017, achieving an accuracy of up to 99.98%. Its benefits include accuracy and robustness, but its drawbacks include complexity and sensitivity to data quality. Future research aims to reduce complexity and improve threat response.

Stefanos Tsimenidis et al.^44^ examined deep learning models for IoT intrusion detection, emphasizing their effectiveness compared to more conventional techniques. Benefits include accuracy and adaptability, but drawbacks include computational demands and data scarcity. Future research proposes effective, decentralized, unsupervised IDS solutions. MINH-QUANG TRAN et al.^45^ introduced an IoT-DNN system for real-time CNC machine monitoring that achieves precise cutting condition categorization and cyberattack detection. Although it has a high degree of reliability, it also presents complexity and environmental issues, and may eventually be utilized in larger innovative systems.

Mohameds. Abdalzaher et al.^46^ examined the roles of machine learning and the Internet of Things in intelligent systems, presenting a taxonomy of machine learning models, assessing security methods, and examining two case studies (Smart Campus and EWS) before suggesting future research avenues. However, it has drawbacks, such as being too general. Martin Manuel Lopez et al.^47^ described an EVL-based IoT intrusion detection system that uses SCARGC to manage concept drift. It has been tested on BotIoT and ToNIoT datasets and has demonstrated good accuracy; nevertheless, it has limitations, such as scalability, and further research is needed to integrate EVL fully.

Vanlalruata Hnamte et al.^48^ report that the two-stage LSTM-AE hybrid IDS presented in this paper achieves an accuracy of up to 99.99% when tested on the CICIDS2017 and CSE-CICIDS2018 datasets. Its benefits include precision and robustness, but its drawbacks include a lengthy training period. Future research will examine attention mechanisms, transfer learning, and ensemble approaches. JIAWEI DU et al.^49^ present the deep learning model NIDS-CNNLSTM, which combines CNN and LSTM for IIoT intrusion detection, in this study. It has been tested on KDDCUP99, NSL-KDD, and UNSW-NB15 datasets and has demonstrated good accuracy and minimal false alarms. Future work aims to address data imbalance and improve small-sample performance.

Privacy and communication-efficient IDS via a federated and collaborative approach offers performance efficiency, but this comes at the cost of sacrificing distributed big data processing and the gradient-based explanation mechanism. Our framework builds on these efforts and integrates distributed processing and interpretable deep learning for IoT.

### Model interpretability and explainability in IDS

Explainable AI techniques have been introduced to interpret the decision-making of black-box IDS models. Approaches such as Grad-CAM, SHAP, LIME, and saliency maps are increasingly used to highlight the features contributing to predictions, thereby fostering trust in AI-based security systems. Tao Yi et al.^50^ examined deep learning-based network attack detection, considering sample imbalance, traffic complexity, and evolving threats. It also identifies current issues and makes recommendations for future research into reliable and comprehensible models.

Sydney Mambwe Kasongo^51^ introduced an IDS framework utilizing XGBoost-based feature selection, which incorporates RNNs (LSTM, GRU, and Simple RNN). Tested on the UNSW-NB15 and NSL-KDD datasets, the results demonstrate faster training times and increased accuracy. Future research will use hybrid approaches.

Ahmed Abdelkhalek and Maggie Mashaly^52^ offered a method for resampling data that addresses class imbalance in NIDS by utilizing Tomek Links and ADASYN. Results from tests on the NSL-KDD dataset demonstrate increased detection rates and accuracy (99.8%). Other datasets will be tested in a future study. Soumyadeep Hore et al.^53^ propose the DeepResNIDS framework, which utilizes AI and transfer learning to identify both known and unknown threats, in this research. When tested on benchmark datasets, its accuracy was 98.5%. Future research will focus on integrating human analysts and effective retraining.

Mamatha Maddu and Yamarthi Narasimha Rao^54^ proposed a deep learning-based intrusion detection system (IDS) for Software-Defined Networking (SDN) that utilizes DCGAN, CenterNet, ResNet152V2, and SMA to identify and prevent network intrusions. It demonstrated good accuracy (99.65% and 99.31%) when tested on the InSDN and Edge IIoT datasets. Future research will focus on the actual installation of IoT networks and the detection of zero-day vulnerabilities. Nojood O. Aljehane et al.^55^ presented GJOADL-IDSNS, a network intrusion detection system that uses SSA for hyperparameter tuning, A-BiLSTM for classification, and GJOA for feature selection. When tested on benchmark datasets, it outperforms current models. Adapting to real-time situations and changing cyber threats is part of the future effort.

Khushnaseeb Roshan et al.^56^ examined adversarial assaults on NIDS using methods such as PGD and FGSM. It exhibits enhanced robustness after testing three defense tactics. Static datasets and streamlined attack models are among the drawbacks. Exploring new attacks and black-box situations is part of future work. Hichem Sedjelmaci^57^ offered a detection method based on hierarchical reinforcement learning for protecting 5G networks from both external and internal threats. Effective detection of unknown assaults and negligible computing overhead are demonstrated. In the future, performance in actual 5G networks will be assessed.

Ahmad Ali AlZubi et al.^58^ presented a cognitive machine learning (ML)-assisted threat detection framework that achieves excellent prediction accuracy (96.5%) and efficiency (97.8%) for safeguarding healthcare cyber-physical systems. In the future, clever CPS security procedures will be developed. SHAKILA ZAMAN et al.^59^ examined IoT security risks and approaches for countermeasures based on AI/ML. It highlights the need for enhanced AI models and improved node security. Future research will concentrate on expanding datasets and increasing efficiency. Celestine Iwendi et al.^60^ introduced an Intrusion Detection System (IDS) for IoT cyberattack detection that uses LSTM classifiers and deep learning. At 99.09% accuracy, it outperformed the most advanced models. Future projects will involve integrating blockchain technology and conducting real-time testing.

### Benchmarking and evaluation using public datasets

Numerous studies benchmark IDS models on datasets such as NSL-KDD, CIC-IDS2017, UNSW-NB15, and TON_IoT. These comparisons help assess generalizability, robustness, and detection accuracy under diverse attack conditions. G.C. Amaizu et al.^61^ proposed a 99.66% accurate composite DDoS detection framework for 5G/B5G networks, which utilizes an effective feature extraction process and a multilayer perceptron. Improving latency and lowering computing cost are the goals of future research. Murat Kuzlu et al.^62^ examined attack methods, defense AI algorithms, and the difficulties in cybersecurity as they relate to IoT, AI, and hostile AI. Future dangers are emphasized, as is the necessity of improved IoT security against changing attacks. Kavitha Dhanushkodi and S. Thejas^63^ examined the application of AI to cybersecurity, emphasizing developments in threat detection, including federated learning and deep learning. Privacy and real-time processing are challenges. Future research will integrate AI with cutting-edge technologies to enhance its capabilities.

Sowmya T. and Mary Anita E. A^64^ examined AI-based intrusion detection techniques, emphasizing ensemble learning, machine learning, and deep learning. The results demonstrate over 99% detection accuracy; future research will focus on identifying unidentified threats and developing hybrid models. Salwa Alem^65^ presented BIANO-IDS, which combines neural networks with anomaly-based and specification-based intrusion detection systems (IDS). It exhibits few false positives and great accuracy when tested in an actual industrial setting. Improving performance metrics is the primary focus of future work.

Heng Zeng et al.^66^ offered a conceptual paradigm for smart city IoT networks that emphasizes user interaction and ongoing cybersecurity education through AI-based anomaly detection. Empirical research and policymakers’ recommendations are part of the future work. Matthew Baker et al.^67^ present an anomaly detection and correction method for power electronic-dominated grids (PEDGs) based on LSTM-MPC in this research. With real-time detection, categorization, and remedial measures, it has been tested on a 14-bus system. Monika Vishwakarma and Nishtha Kesswani^68^ introduced a deep neural network-based intrusion detection system (IDS) for Internet of Things (IoT) networks that can identify 20 different types of attacks using a benchmark dataset. The results demonstrate high efficiency, and real-time training will be the primary focus of future research.

Md. Asaduzzaman and Md. Mahbubur Rahman^69^ introduced a hybrid LSTM-CNN model that improves accuracy to 93.53% for zero-day attack detection utilizing GAN-generated data. To detect more complex attacks, future studies will expand the scope of attack data. ZHIBO ZHANG et al.^70^ discuss the necessity for transparency in AI-driven defense models in this paper’s evaluation of Explainable AI’s (XAI) application in cybersecurity. For XAI applications, it outlines a research path, obstacles, and future objectives.

Ankit Attkan and Virender Ranga^71^ examined IoT security with an emphasis on session keys, blockchain-AI integration, and authentication. It draws attention to issues like battery life and offers AI-powered solutions for IoT key management. Future studies are required. S. Markkandeyan et al.^72^ propose a hybrid Deep Learning model (ATFDNN + IPSO) in the study to identify malware and software piracy in the Internet of Things. Experiments on the Maling and Google Code Jam datasets demonstrate that it outperforms traditional techniques. Reliance on massive datasets and significant computational expense are among its drawbacks. Qawsar Gulzar and Khuram Mustafa^73^ present a DeepCLG hybrid learning model for IIoT network intrusion detection in the research; it achieves high accuracy (99.82%) on the UNSW_NB15 and CICIoT 2023 datasets. Scalability and real-time flexibility are limitations that warrant further study.

### Optimization techniques and hybrid model designs

Recent trends in IDS research include ensemble learning, neural architecture optimization, hyperparameter tuning, and hybrid deep learning architectures that combine CNNs, RNNs, or attention mechanisms for improved performance. Enerst Edozie et al.^74^ examined AI-powered anomaly detection for telecom networks, emphasizing the efficiency of deep learning. Latency, scalability, and data volume are obstacles. Future research should focus on ongoing model maintenance and the development of hybrid models. GIOVANNI BATTISTA GAGGERO et al.^75^ examined anomaly identification in smart grids with an emphasis on physics- and AI-based techniques. It identifies false favorable rates and real-world testing gaps, suggesting further study to increase grid dependability.

Vivek Menon et al.^76^ examined the importance of AIoT in new technologies, architecture, and security. To direct future research in AIoT applications and advances, this addresses ML/DL algorithms, IoT security strategies, and associated problems. Malka N. Halgamugea and Dusit Niyato^77^ introduced an AI/ML-integrated adaptive edge security architecture for the Internet of Things that generates dynamic policies. Addressing issues in IoT ecosystems improves compliance and resiliency. Validation and optimization are part of future development. Ilhan Firat Kilincer^78^ uses the CL2-IDS dataset in the study to demonstrate a hybrid deep learning model (CNN + Bi-LSTM) for identifying Layer 2 network attacks. Its accuracy was 95.28%. Future research will focus on enhancing anomaly detection and growing the dataset.

Huiyao Dong and Igor Kotenko^79^ examined machine learning methods in IDS, contrasting deep learning strategies with conventional machine learning models. It draws attention to developments, difficulties, and upcoming studies, especially in hybrid models and Internet of Things settings. Moneerah Alotaibi et al.^80^ introduced a hybrid intrusion detection system (IDS) model that uses bio-inspired metaheuristic algorithms (GWQBBA) for attack categorization and feature selection. Random Forest reduces the number of features to 12 and achieves an accuracy of 98.5%. Enhancing scalability and optimizing parameters are the primary goals of future studies.

**Fig. 1:**
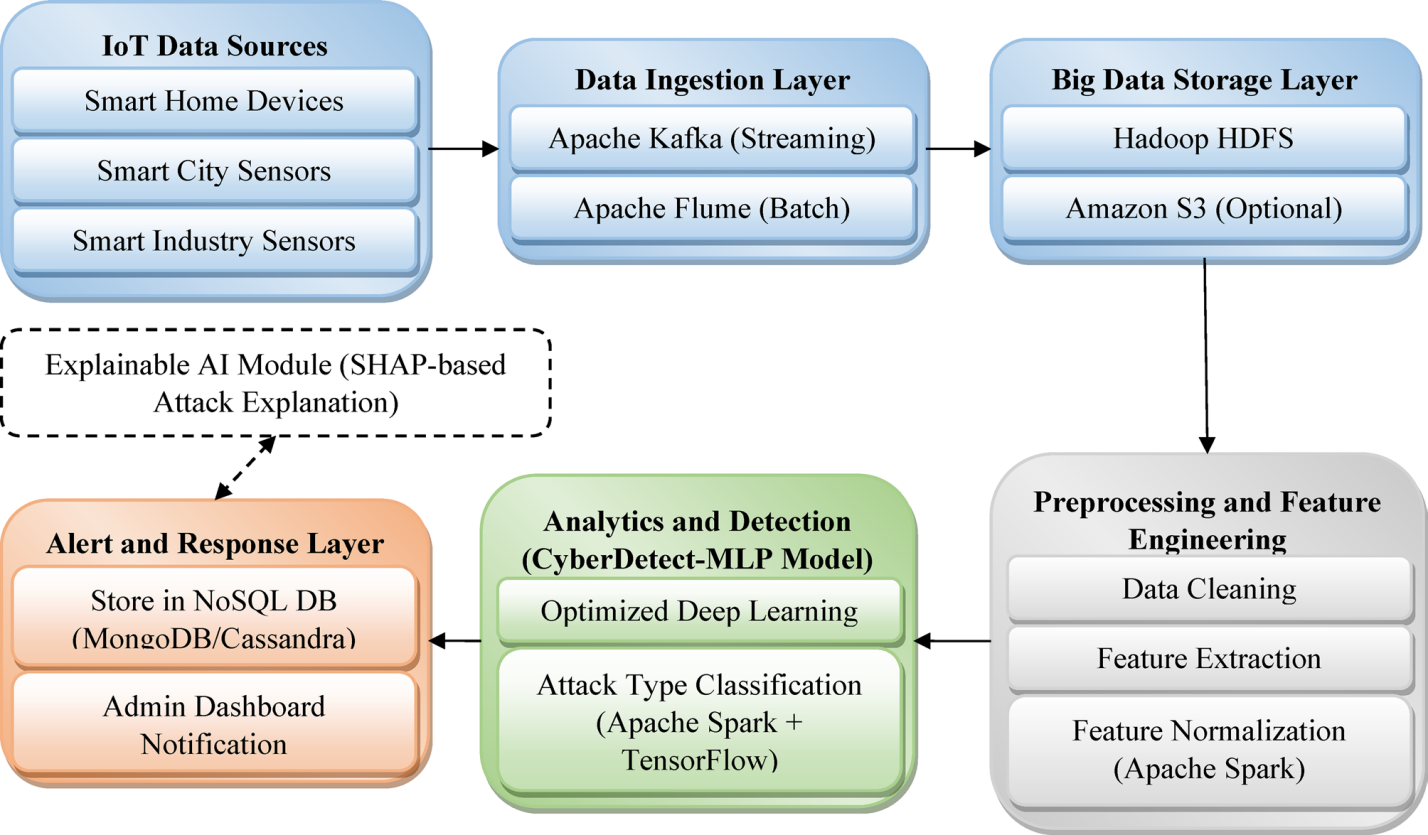
system architecture of the proposed big data-enabled cyberattack detection framework using CyberDetect-MLP for IoT environments.

Existing research has investigated enhanced strategies for IoT intrusion detection and threat prediction, with a focus on explainability and scalability, as well as with emerging AI approaches. Korba et al. An early work that proposed an explainable anomaly detection model for early-stage IoT botnet attack mitigation^81^, whereas Korba et al. To this end, a zero-day botnet detection approach using an Isolation forest and Particle Swarm Optimization was developed by^82^ (highlighted with a blue block in Fig. 1). Further, Korba et al. have introduced a complete AI model-based framework for rapid Internet of Things botnet detection through network traffic analysis in^83^. Diaf et al. Labiod et al. utilize large language models for predictive analysis of cyber-attacks in IoT networks, as seen in^84,85^. An IDS architecture is designed to address the IoT security challenge in a fog-based infrastructure^86^. Khan et al. Khan et al.^87^ proposed a privacy-preserving, federated boosting technique to amplify attack detection capabilities through collaboration among distributed IoT devices. Lastly^88^, presented a network aimed at collaborative SRU with both behavior aggregation and interpretability. Although these studies highlight advancements, they lack the unification of a significant data processing stream^8^ with integrated explainable deep learning^13^, which is addressed in our work.

In the past few years, deep learning based techniques have shown great promise in enabling IoT and IIoT intrusion detection. Research on IDS based on deep learning^89^ in industrial IoT environments showed promising accuracy results using optimized neural architectures. Likewise, TL-BILSTM IoT proposed a transfer learning model to improve the performance of IDSs by utilizing bidirectional LSTM layers for IoT networks^90^. For example, ref^91^. proposed an explainable deep learning framework for Industry 5.0 cyber-physical systems and underlined the importance of interpretability for critical infrastructure security. Additionally, a deep learning-based IoT security framework for intrusion detection and prevention^92^ demonstrated that adapting and scaling IDS solutions are necessary.

A variety of machine learning and deep learning models have been investigated, including decision trees, support vector machines (SVMs), convolutional neural networks (CNNs), recurrent neural networks (RNNs), and hybrid ensembles^6^. These methods are undoubtedly promising, but when applied to large-scale, heterogeneous IoT data, they often suffer from the challenges of real-time performance, scalability, and interpretability. Only a handful of works combine big data platforms such as Spark and explainable AI, indicating the necessity for the unified, scalable, and interpretable framework proposed herein.

## Proposed framework

This section presents the proposed CyberDetect-MLP framework, designed to enable scalable and explainable detection of cyberattacks in IoT environments. It details the system architecture, dataset preparation, significant data processing pipeline, optimized deep learning model, algorithmic implementation, and deployment strategy, supported by corresponding figures, algorithms, and tables to illustrate the operational workflow and novel methodological contributions.

### System overview

The overall system architecture for IoT cyberattack detection is designed as a comprehensive big data analytics framework that integrates scalable data ingestion, distributed storage, preprocessing, and an optimized deep learning model for accurate attack classification. IoT telemetry data, network traffic, and operating system logs are continuously collected from diverse sources, including smart homes, cities, and industrial environments. These high-volume and high-velocity data streams are ingested using Apache Kafka and Apache Flume into the Hadoop Distributed File System (HDFS) for fault-tolerant, scalable storage. Apache Spark facilitates distributed preprocessing and feature engineering, enabling efficient handling of data cleansing, normalization, and selection across large datasets. The processed data is then fed into the CyberDetect-MLP model, an optimized multilayer perceptron designed for scalable and accurate cyberattack classification. Detection results trigger real-time alerts and are logged for further analysis. The entire workflow emphasizes scalability, robustness, and real-time responsiveness, making it suitable for dynamic and heterogeneous IoT environments. The overall system architecture is illustrated in Fig. 1.

Figure 1 depicts the high-level architecture of the proposed big data-enabled IoT cyberattack detection framework. It illustrates the seamless integration of IoT data sources with distributed data ingestion mechanisms, scalable storage, and parallel preprocessing components. The diagram illustrates how processed feature sets are integrated into the CyberDetect-MLP model for attack classification, followed by alert generation and storage of detection outcomes. Key elements, including Apache Kafka, Hadoop HDFS, Apache Spark, and an optimized deep learning model, are represented, highlighting the modular and scalable nature of the system. Table 1 summarizes the key symbols and their descriptions used in the methodology to facilitate a clear understanding of the proposed framework.

**Table 1:** Notations and definitions used in the proposed CyberDetect-MLP framework.

Symbol	Description
$\:X$	Original feature value
$\:{X}^{{\prime\:}}$	Normalized feature value after Min-Max scaling
$\:{X}_{min}$	Minimum value of a feature
$\:{X}_{max}$​	Maximum value of a feature
$\:{h}^{\left(l\right)}$	Output of the $\:{l}^{th}$ hidden layer
$\:{h}^{\left(l-1\right)}$	Input to the $\:{l}^{th}$ hidden layer
$\:{W}^{\left(l\right)}$	Weight matrix of the $\:{l}^{th}$ layer
$\:{b}^{\left(l\right)}$	Bias vector of the $\:{l}^{th}$ layer
$\:\sigma\:\left(\bullet\:\right)$	Activation function (ReLU)
$\:{z}_{j}$	Logit output for class j before softmax activation
$\:K$	Total number of classes (attack types)
$\:P\left(y=j\:|\:x\right)$	Predicted probability of class j for input $\:x$
$\:{y}_{ij}$	True label indicator for instance i and class j
$\:{\widehat{y}}_{ij}$	Predicted probability for instance iii and class j
$\:{\theta\:}_{t}$	Model parameter at iteration t
$\:\alpha\:$	Learning rate
$\:{\widehat{m}}_{t}$	Bias-corrected first moment estimate (mean of gradients)
$\:{\widehat{v}}_{t}$	Bias-corrected second moment estimate (uncentered variance)
$\:\epsilon\:$	Small constant to avoid division by zero in optimization updates
$\:{\alpha\:}_{t}$	Learning rate at epoch t in cosine annealing schedule
$\:{\alpha\:}_{min}$​	Minimum learning rate
$\:{\alpha\:}_{max}$	Maximum learning rate
$\:T$	Total number of training epochs in the learning rate schedule

### Dataset description and preparation

In this study, experiments were conducted on the TON_IoT dataset, a comprehensive and large-scale dataset specifically designed for cyberattack detection in the IoT environment. This dataset comprises telemetry data from IoT devices, PCAP/CSV format network traffic captures, and network logs from operating systems such as Windows, Ubuntu, and Android. Such a variety of sources emulates the actual smart home, city, and industrial IoT ecosystems. The TON_IoT dataset consists of subsets, each with a mix of benign activities and cyberattack activities, specifically Denial-of-Service (DoS), Distributed Denial-of-Service (DDoS), ransomware, backdoor access, injection attacks, cross-site scripting, and reconnaissance attack types.

The original TON_IoT data are placed in a Hadoop Distributed File System (HDFS) for scalable storage and concurrent access. For batch ingestion, it uses Apache Flume, while Apache Kafka is used to simulate streams in real-time. The first preprocessing step is to clean the data. Missing values are handled using mean imputation for continuous features and mode imputation for categorical attributes. To maintain the integrity of the data and avoid model bias, duplicate entries are dropped. One hot encoding technique is used for encoding categorical variables like device type or service name. We perform Min–Max normalization on all continuous numerical features to scale the data between 0 and 1 as shown in Eq. 1.

1 $$\:{X}^{{\prime\:}}=\frac{X-{X}_{min}}{{X}_{max}-{X}_{min}}$$

Indeed, $\:X$ is the original value of the feature, $\:{X}_{min}\:$and $\:{X}_{max}$ denote the minimum and maximum value of the feature, and $\:{X}^{{\prime\:}}$ is the normalized value from feature. A process to select the best features is included to enhance the quality of input data and to decrease dimensionality. We use Mutual Information (MI) to pick the top-k most informative features that are the most dependent on the attack labels. The mutual information of a feature X with the target class Y is calculated as in Eq. 2.

2 $$\:I\left(X;Y\right)=\sum\:_{x\in\:X}\sum\:_{y\in\:Y}p\left(x,y\right)\text{log}\frac{p\left(x,y\right)}{p\left(x\right)p\left(y\right)}$$

where $\:p\left(x,y\right)\:\:$the joint probability distribution of both X and Y; and $\:p\left(x\right)$ and $\:p\left(y\right)\:$the marginal probability distribution of X and Y. Features with maximum mutual information scores are kept for model training.

After preprocessing, filtering features and size, the dataset is split in training (70%), validation (15%) and test (15%) sets. Stratified sampling maintains the distribution of the classes in each of these subsets which will help eliminate any imbalance-induced bias affecting the evaluation of the model. The output processed dataset acts as input for training and testing the proposed CyberDetect-MLP model based on the distributed big data analytics framework.

### Big data infrastructure and processing pipeline

To handle the large volume, velocity, and variety of the TON_IoT dataset, the proposed framework adopts a distributed big data infrastructure integrating Hadoop and Apache Spark. The Hadoop Distributed File System (HDFS) is utilized for scalable, fault-tolerant storage of ingested IoT telemetry, network traffic, and operating system log data. This enables high-throughput access across distributed nodes, ensuring that large-scale datasets can be efficiently read and written without centralized bottlenecks.

Data ingestion into the big data environment is carried out through two primary mechanisms. Apache Kafka serves as the real-time ingestion layer, simulating continuous IoT telemetry and network traffic streams. Apache Flume is used for batch ingestion of historical datasets, ensuring compatibility with structured CSV and semi-structured log data formats. The ingested data is serialized into efficient storage formats such as Avro or Parquet before being placed in HDFS to enhance compression and parallel processing capabilities.

Once stored, the data undergoes distributed preprocessing and feature engineering using Apache Spark. Spark’s Resilient Distributed Dataset (RDD) abstraction and DataFrame API enable parallel execution of data cleaning, missing value imputation, duplicate removal, categorical encoding, and feature normalization. Continuous features are normalized between 0 and 1 using the Min-Max normalization technique described previously in Eq. (1), ensuring uniform feature scaling for stable deep learning model convergence.

**Fig. 2:**
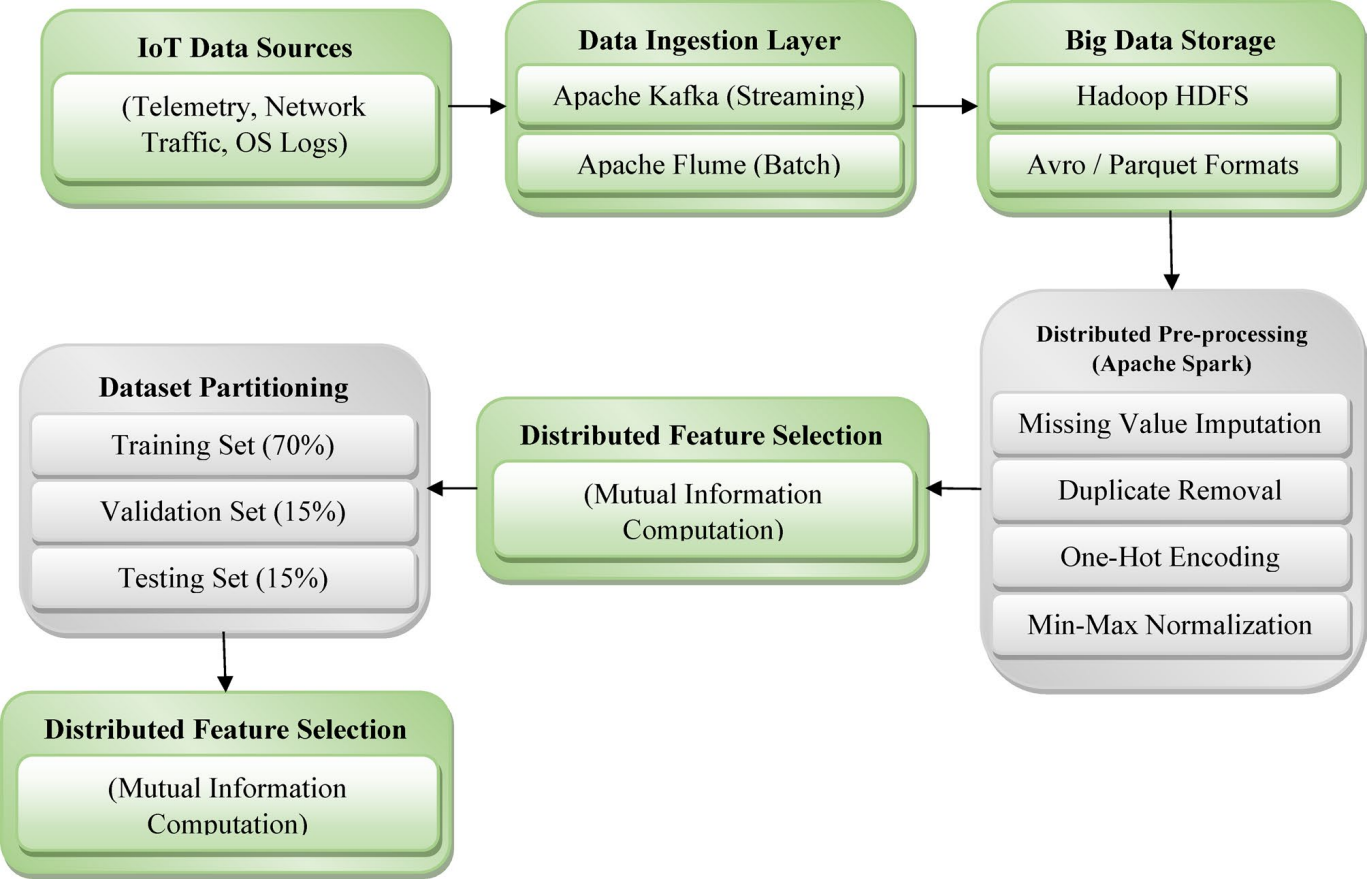
Big data infrastructure and processing pipeline for IoT cyberattack detection.

Figure 2 illustrates the distributed pipeline for data ingestion, storage, preprocessing, and feature selection using Hadoop and Apache Spark. For feature selection, Spark’s scalable computation is leveraged to calculate Mutual Information (MI) scores between input features and the target labels. Using the scoring method that has been defined and described in Eq. (2), features are rank-ordered, and the top-k most informative features are selected. Mason can carry out these computations in a distributed manner, allowing feature engineering that would be too computationally intensive otherwise to run on larger-scale IoT datasets.

After feature engineering, the data is partitioned into training, validation, and testing subsets using stratified sampling to maintain attack type class distributions across splits. This preprocessed and feature-selected data is subsequently used as input for the training and evaluation of the CyberDetect-MLP model. The integrated big data infrastructure ensures scalability, real-time data adaptability, and high computational efficiency, critical for IoT cyberattack detection applications.

### Proposed CyberDetect-MLP model architecture

**Fig. 3:**
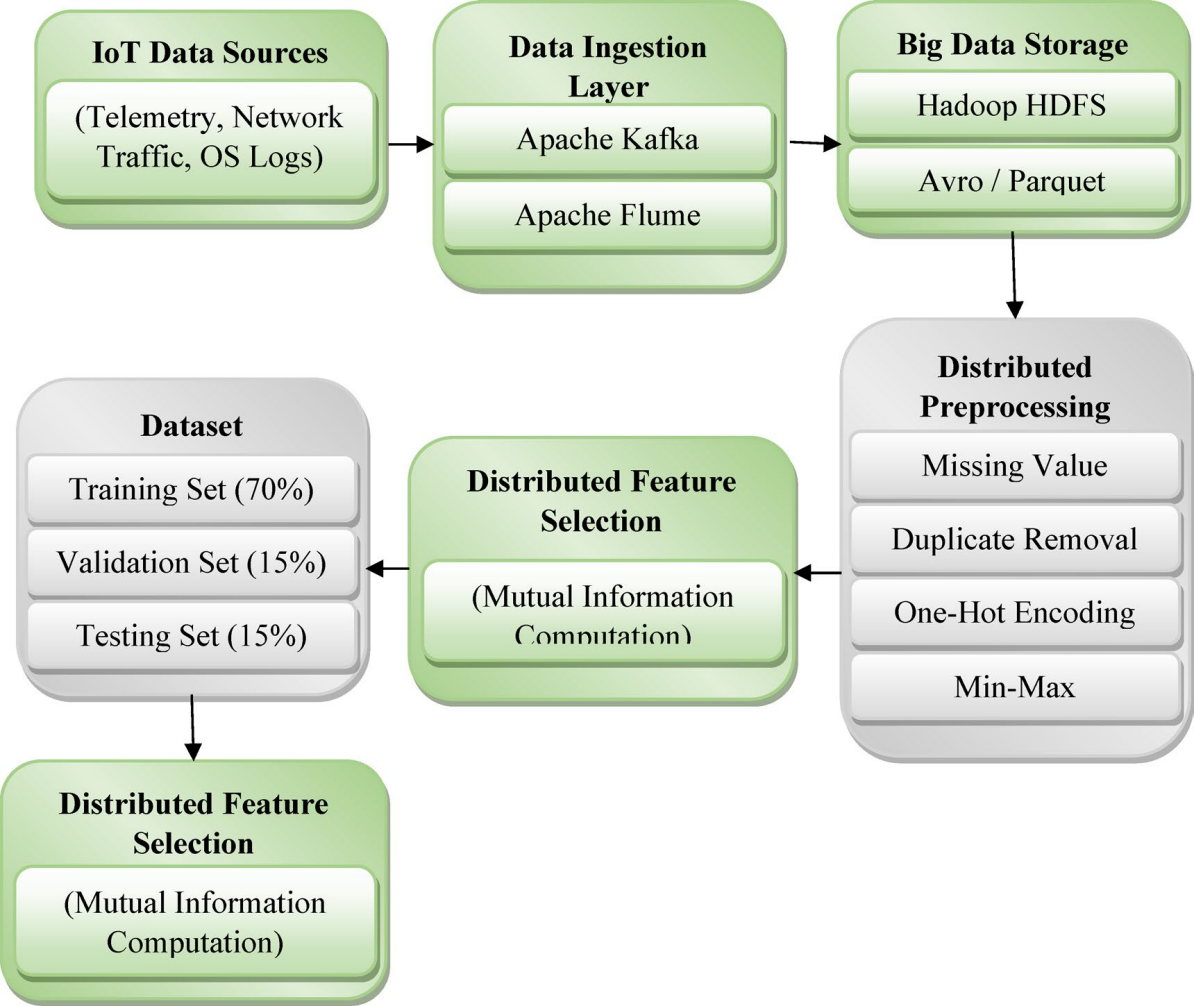
Architecture of the proposed CyberDetect-MLP model for scalable IoT cyberattack detection.

At the heart of the proposed framework is CyberDetect-MLP, shown in Fig. 3, which is a highly optimized deep learning architecture for scalable cyberattack detection in IoT environments. The input to the model is the dataset based on the TON_IoT sources that is feature-selected and normalized. The input vectors correspond to instances of telemetry data, network traffic, or system logs, and are already pre-processed to maintain only the features relevant to the attack classification task.

The preprocessed feature set is passed as input to CyberDetect-MLP through its input layer and is followed by one or more fully connected dense layers. The initial dense layer contains 512 neurons with ReLU activation for non-linearity, and after that uses batch normalization to stabilize and speed up the learning. Preventing Overfitting: Dropout layer rate = 0.3. Dropout is used to prevent overfitting by randomly turning off neurons during training. We pass the output of the first hidden layer to the second dense layer with 256 neurons, again with ReLU activation, batch normalization, and dropout. Then, it is followed by a third dense layer with 128 neurons. The transformation at each of the dense layers $\:l$ can be expressed mathematically as in Eq. 3.

3 $$\:{h}^{\left(l\right)}=Dropout\left(BatchNorm\left(\sigma\:\left({W}^{\left(l\right)}{h}^{\left(l-1\right)}+{b}^{\left(l\right)}\right)\right)\right)$$

Where $\:{h}^{\left(l-1\right)}$ is the input of the previous layer,$\:\:{W}^{\left(l\right)}$ and $\:{b}^{\left(l\right)}$ are the weights and biases of the current layer, $\:\sigma\:\left(\bullet\:\right)$ is the ReLU activation function, and batch normalization and dropout are sequentially applied.

In the last stage, the output layer is a dense layer with as many neurons as the number of attack classes. Multi-class attack classification is performed using a softmax activation function to produce normalized probability distributions per class. A single output neuron softmax operation for an output neuron $\:j\:$ is defined as in Eq. 4.

4 $$\:P\left(y=j\:|\:x\right)=\frac{{e}^{{z}_{j}}}{{\sum\:}_{k-1}^{K}{e}^{{z}_{k}}}\:\:\:\:\:\:\:\:\:\:\:\:\:\:\:\:\:\:\:\:\:\:\left(4\right)$$

K is the number of classes, $\:{z}_{j}$ is the logit for clas j. Due to multiclass classification nature, the model is trained with a categorical cross-entropy loss that inflicts a penalty on wrong class predictions while ensuring maximizing the probability of correct class, at the same time. L = loss function over N instances as in Eq. 4.

Where $\:{y}_{ij}$ is the indicator (1 or 0) if class label c is the correct classification for observation and $\:{\widehat{y}}_{ij}$ is the predicted probability of class j. Finally, to improve the efficiency and the generalization of the training, a learning rate scheduler (cosine annealing, step decay) is added. This progressively reduces the learning rate over the training epochs to enable the model to converge faster and escape local minima traps. Figure 3 provides a human figurative illustration of the CyberDetect-MLP architectural design, such as Input, Hidden, and Output layers, and optimization mechanisms.

### Model training and hyperparameter optimization

Trained the supervised model CyberDetect-MLP on the selected features from the preprocessed TON_IoT dataset. This categorical cross-entropy loss described in Eq. (7) is minimized during the training to enable the model to successfully classify the various types of cyberattacks encountered in the IoT environments. Using stratified sampling to ensure the distribution of attack classes is maintained within the subsets, the dataset is then split into a training, a validation, and a test sample in a 70:15:15 ratio, respectively.

We perform mini-batch gradient descent over epochs for training. We define a batch size to ensure every batch contains a fixed number of samples. We then update the model weights after each step (batch) using Adam. Adam, which combines the advantages of both AdaGrad and RMSProp. Adams supplies adaptive learning rates for each parameter to speed up convergence. We can update a parameter θ at iteration tt using Adam as in Eq. 5.

5 $$\theta _{t} = \theta _{{t - t}} - \alpha \frac{{\hat{m}_{t} }}{{\sqrt {\hat{v}_{t} } + \smallint }}$$

where α is the learning rate,, $\:{\widehat{m}}_{t}$ and $\:{\widehat{v}}_{t}$ are the bias-corrected moving averages of the first and second moments of the gradients, and ϵ is a small constant (usually 10e-8) as a condition against zero for the denominator.

Hyper-parameter tuning is used to improve the CyberDetect-MLP performance. Hyperparameters tuned in this model include number of hidden layers, number of neurons per layer, drop out rates, batch size, initial learning rate and learning rate decay schedule. It is actually a randomized search within pre-defined ranges of the parameters so it is faster and less computationally expensive than an exhaustive search. In the case, each hidden layer dropout number of neurons and each dropout rates were tested as in the range providing in the [128, 256, 512] as well [0.2, 0.3, 0.5].

Moreover, a learning rate scheduler is added during training. Cosine annealing schedule (Loshchilov & Hutter 2016) gradually decreases the learning rate according to a cosine function, which enables the model to quickly learn during the initial stages of training while also fine-tuning during the later stages. Cosine annealing is one of the popular scheduling techniques, and the learning rate $\:{\alpha\:}_{t}$ at epoch t under cosine annealing can be expressed as in Eq. 6.

6 $$\:{\alpha\:}_{t}={\alpha\:}_{min}+\frac{1}{2}\left({\alpha\:}_{max}-{\alpha\:}_{min}\right)\left(1+\text{cos}\left(\frac{t\pi\:}{T}\right)\right)$$

With $\:{\alpha\:}_{min}$ and $\:{\alpha\:}_{max}$ as the minimum and maximum learning rates respectively and T as total number of training epochs. We use standard classification metrics to evaluate model performance, including accuracy, precision, recall, F1 score, and area under the ROC curve (ROC-AUC). These metrics offer a holistic evaluation of the CyberDetect-MLP detection ability under both balanced and imbalanced attacks. The hyperparameter configurations that result in the highest validation F1-score are used as the final parameters of the model, which is then evaluated on test data and/or used for deployment.

The TON_IoT dataset is heavily imbalanced, with a very low number of attacks of a specific type. In an attempt to mitigate this, we used several approaches: (i) class-weighted categorical cross-entropy loss, with weights inversely proportional to class frequencies, and (ii) some mild oversampling of the minority classes during training to improve representation. Such techniques enable the model to learn discriminative features for minority classes while avoiding overfitting.

### Algorithmic implementation

This section presents the algorithmic implementation of the proposed framework, detailing the core computational procedures that enable scalable and efficient cyberattack detection in IoT environments. This section outlines the step-by-step algorithms for data preprocessing, feature selection, model training, and real-time detection. By formalizing these processes, the framework ensures reproducibility, clarity, and systematic execution, facilitating seamless integration of big data analytics and optimized deep learning techniques for robust IoT security.

**Algorithm 1:**
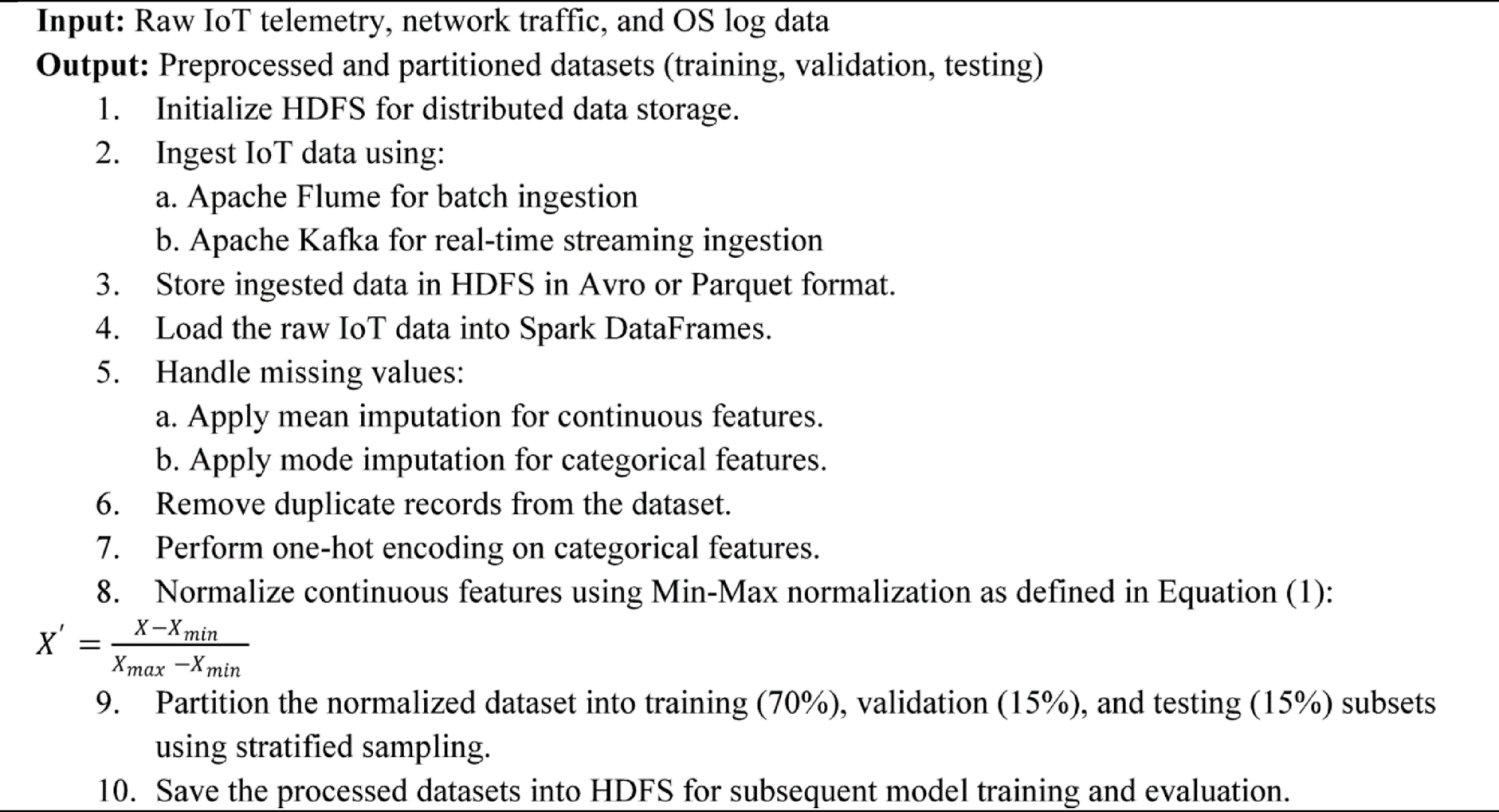
Big Data Preprocessing and Ingestion Pipeline.

Algorithm 1 outlines the end-to-end big data preprocessing and ingestion pipeline essential for preparing IoT data for effective cyberattack detection. It details the processes of scalable data storage initialization using Hadoop HDFS, real-time and batch data ingestion through Apache Kafka and Flume, and the conversion of raw data into efficient storage formats. The algorithm further describes distributed data cleaning operations, including missing value imputation and duplicate removal, categorical feature encoding, and normalization of continuous features. Finally, it partitions the processed dataset into training, validation, and testing subsets, ensuring balanced class distributions for reliable model training and evaluation. This pipeline provides the large-scale, heterogeneous IoT data is transformed into a consistent and optimized format suitable for subsequent analytics.

**Algorithm 2:**
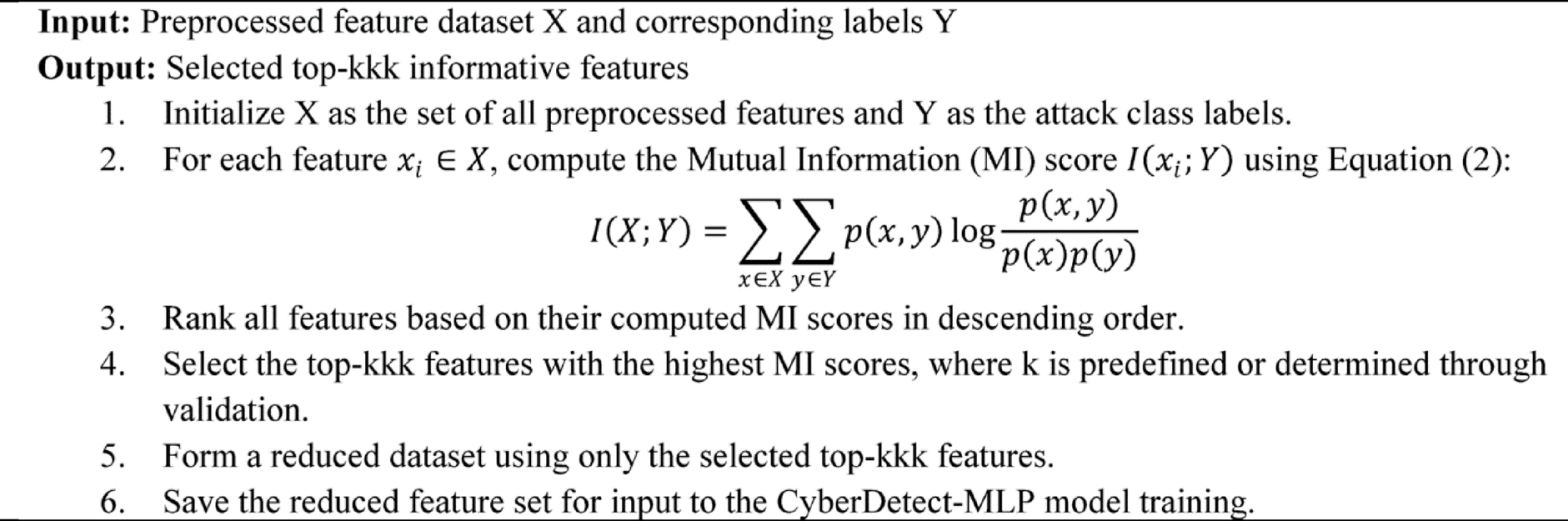
Feature Selection Using Mutual Information.

Algorithm 2 describes the feature selection process using Mutual Information to identify the most informative features for cyberattack detection. It takes the preprocessed dataset and corresponding class labels as input and computes the Mutual Information score for each feature, quantifying its dependency with the target variable. Features are then ranked based on their scores, and the top-kkk features are selected to reduce dimensionality while preserving predictive power. This step enhances model efficiency and accuracy by focusing training on the most relevant attributes, thereby improving the performance of the CyberDetect-MLP model.

We measure the relevance of these features for IoT intrusion detection using a mutual-information (MI) based feature selection procedure to reduce their high dimensionality. During every cross-validation fold or train–test split, MI scores are only calculated on the training subset to avoid any risk of information leakage. Subset of features selected from training data are then used on the corresponding test samples. It guarantees that you are not peeking at the test set by having any of the test set information influence the feature selection process, keeping the evaluation clean.

**Algorithm 3:**
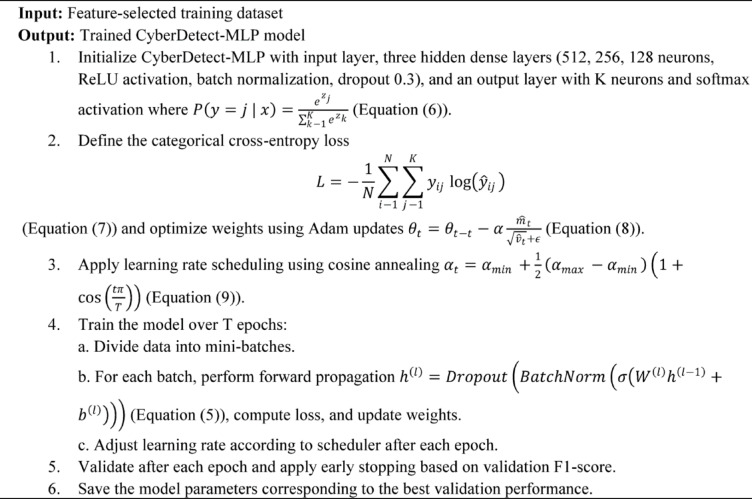
CyberDetect-MLP model training and optimization.

Algorithm 3 details the training and optimization process of the CyberDetect-MLP model. Starting with the initialization of a multilayer perceptron architecture incorporating dense layers, ReLU activations, batch normalization, and dropout regularization, the algorithm employs the categorical cross-entropy loss function optimized via the Adam optimizer. A learning rate scheduler based on cosine annealing dynamically adjusts the learning rate during training to facilitate efficient convergence. Training proceeds over multiple epochs with mini-batch gradient descent, where forward propagation computes layer outputs and backpropagation updates model weights. Validation after each epoch guides early stopping to prevent overfitting, and the best-performing model parameters are saved for deployment. This algorithm ensures scalable and accurate cyberattack classification within the big data framework.

**Algorithm 4:**
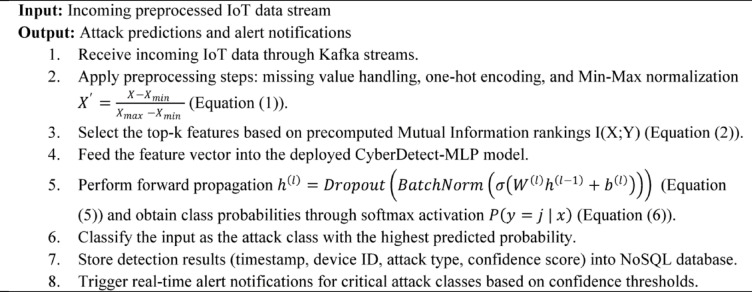
Real-time cyberattack detection and alert generation.

Algorithm 4 describes the real-time cyberattack detection and alert generation process within the deployed framework. It begins by receiving preprocessed IoT data streams, which are normalized and feature-selected based on prior computations. The data is then passed through the trained CyberDetect-MLP model for inference, where forward propagation produces class probabilities for attack types. The algorithm classifies inputs by selecting the highest probability class and logs detection results, including timestamps and confidence scores, into a scalable NoSQL database. Critical detections trigger immediate alerts to system administrators, enabling timely responses. This algorithm ensures continuous, low-latency threat monitoring and effective incident management in dynamic IoT environments.

The proposed CyberDetect-MLP framework enables end-to-end intrusion detection via a structured pipeline: distributed data ingestion, pre-processing, and feature engineering based on Spark, mutual information–based feature selection, and optimized MLP training with batch normalization, dropout, and cosine annealing scheduling, as well as real-time inference with XAI-based interpretation (Grad-CAM and SHAP). To facilitate the understanding of the overall workflow, each algorithmic stage is explained — along with the associated steps.

The computational complexity of the framework is linear with respect to the dataset size n and feature dimension m. Spark preprocessing and feature selection require O(n⋅m) time, distributed across worker nodes. For the MLP, forward and backward propagation have a cost of $\:O\left({\sum\:}_{i=1}^{L}{h}_{i-1}\:.\:{h}_{i}\right)$, where L is the number of layers and h_i_​ are neurons per layer; space complexity is of the same order. Inference per sample is lightweight, enabling near real-time detection. This analysis confirms that CyberDetect-MLP scales efficiently for large-scale IoT deployments.

### Evaluation methodology

The systematic evaluation of cyberattack detection performance on the TON_IoT dataset with the proposed CyberDetect-MLP model. For a robust and fair assessment, we perform stratified sampling such that the distribution of attack classes within train, validation, and test sets is proportional to that in the dataset. To avoid any data leakage and to simulate the deployment condition, the testing set is never seen during the training and hyperparameter tuning phases of any model.

The performance metrics chosen for evaluation are accuracy, precision, recall, F1-score, and ROC curve (the area under the Receiver Operating Characteristic curve). Accuracy provides a better understanding of the model’s tendency, indicating how accurate the model is overall. This is achieved through the equation given in Eq. 7.

7 $$\:Accuracy\:=\frac{TP+TN}{TP+TN+FP+FN}$$

where TP is true positives, TN true negatives, FP is false positives, FN is false negatives. Precision measures the number of true positive predictions out of all positive predictions made, which can be defined as in Eq. 8.

8 $$\:Precision\:=\:\frac{TP}{TP+FP}$$

Recall, which is also known as sensitivity, or true positive rate, is the ratio of correctly predicted positive observations to actual positives and is defined as in Eq. 9.

9 $$\:Recall\:=\:\frac{TP}{TP+FN}$$

The F1-score, which strikes a balance between precision and recall, considers false positives and false negatives, and is defined as in Eq. 10.

10 $$\:F1-score=2\times\:\frac{\:Precision\times\:\:Recall\:}{\:Precision+\:Recall\:}$$

The other metric, ROC-AUC, measures the model’s capacity to separate classes with different thresholds. The greater the AUC, the more reducible the attack and normal instances are.

Evaluation of the testing set for all metrics has been taken into account to verify the generalization ability of CyberDetect-MLP. To better evaluate the performance of the proposed model, we also compare it with baseline models, where their Random Forest and XGBoost models are trained on the same dataset and preprocessing pipeline. We present a comparative report of performance across all chosen metrics, proposing an optimal deep-learning framework. Based on this, we justify the advantages of our approach in terms of detection accuracy, robust handling of class imbalance, and scalability for large-scale IoT-based cybersecurity applications.

### Deployment strategy and alert generation

After training and validating the CyberDetect-MLP model, the framework proceeds to deployment for real-time cyberattack detection and alert generation within IoT environments. The deployment pipeline integrates the trained model into the distributed significant data infrastructure to ensure scalability, low-latency inference, and seamless integration with existing IoT systems.

The incoming IoT data streams, either simulated through Apache Kafka or sourced from real-time telemetry devices, are first preprocessed in a manner consistent with the training pipeline. This ensures that feature distributions remain uniform, avoiding inference drift. The preprocessed feature vectors are passed to the deployed CyberDetect-MLP model for real-time classification. Given the lightweight architecture of the model and optimization through feature selection, inference is achieved within millisecond latency, making it suitable for rapid attack identification in dynamic IoT environments.

Detection results, consisting of predicted attack classes and corresponding confidence scores, are stored in a distributed NoSQL database such as MongoDB or Apache Cassandra. This enables scalable logging of cybersecurity events, allowing retrospective analysis and auditing. Each detected attack instance triggers an automated alert mechanism that updates an administrator dashboard with incident details, including device ID, attack type, timestamp, and severity score. For critical attacks such as ransomware or DDoS, the system is configured to generate high-priority alerts, optionally integrating with existing network management systems for automated containment actions.

Furthermore, an optional explainable AI (XAI) module can be integrated to provide human-interpretable reasons behind each classification decision. Using SHAP (Shapley Additive exPlanations) values, the system can highlight the most influential features contributing to a specific attack prediction. This enhances administrator trust and supports informed incident response planning. The combined deployment of CyberDetect-MLP within a distributed big data framework ensures that the system can maintain high detection accuracy, rapid response times, and scalable performance even as the volume and velocity of IoT data continue to increase.

## Experimental results

This section reports the experimental evaluation of the proposed CyberDetect-MLP framework using the full TON_IoT dataset^93^. It includes the setup configuration, performance comparison with baseline models, visualization-based analysis, ablation study, and explainability validation. The results demonstrate the framework’s high accuracy, scalability, and interpretability, highlighting its effectiveness for real-world IoT cyberattack detection scenarios.

### Experimental setup

The experimental setup is designed to evaluate the effectiveness, scalability, and reproducibility of the proposed CyberDetect-MLP framework for detecting IoT cyberattacks. All experiments are conducted on a distributed computing environment comprising a five-node Hadoop-Spark cluster. Each node is configured with Intel Xeon processors, 64 GB of RAM, and Ubuntu 20.04 LTS, and is connected over a high-speed Gigabit Ethernet network. Apache Hadoop 3.3.2 and Apache Spark 3.4.0 are deployed for storage and distributed processing. The CyberDetect-MLP model is implemented using TensorFlow 2.13 integrated with PySpark for seamless data loading and training across the Spark environment. NoSQL storage and alert modules are deployed using MongoDB 6.0, while Apache Kafka 3.5 is used to simulate real-time data streams.

The prototype application workflow starts by ingesting data from the TON_IoT dataset, including telemetry, network, and OS log data, into HDFS. Ingestion is handled via Kafka topics (for simulated real-time input) and Flume agents (for batch logs). All preprocessing steps, including null value imputation, categorical encoding, and Min-Max normalization, are executed using Spark DataFrames and written back to HDFS for training. Feature selection is performed using Spark’s Mutual Information feature selector, and the top 30 features are retained based on empirical validation. Table 2 summarizes the experimental setup, including the dataset, tools, cluster configuration, and hyperparameter settings to ensure reproducibility and scalability.

**Table 2:** Experimental setup summary for CyberDetect-MLP framework.

Component	Specification/ description
Dataset	TON_IoT (Telemetry, Network Traffic, OS Logs)
Data ingestion tools	Apache Flume (batch), Apache Kafka (real-time simulation)
Storage framework	Hadoop Distributed File System (HDFS)
Processing framework	Apache Spark 3.4.0
Machine learning library	TensorFlow 2.13 is integrated with PySpark.
Cluster configuration	5 nodes, Intel Xeon CPUs, 64 GB RAM per node, Ubuntu 20.04, Gigabit Ethernet
Feature selection method	Mutual Information (top-30 features selected)
NoSQL storage	MongoDB 6.0
Alert module	Real-time alerts via the admin dashboard and Kafka trigger-based notifications
Hyperparameter ranges	Neurons: {128, 256, 512}, Dropout: {0.2, 0.3, 0.5}, Batch Size: {64, 128}, LR: {1e-3, 5e-4, 1e-4}
Learning rate scheduler	Cosine annealing
Epochs/early stopping	Max 100 epochs, early stopping with patience = 10
Reproducibility strategy	Random seed, three repeated runs, tuning scripts, complete logs, and configurations retained

To ensure replicability, hyperparameter tuning is carried out using randomized search over the following ranges: number of neurons per dense layer in {128, 256, 512}, dropout rates in {0.2, 0.3, 0.5}, batch sizes in {64, 128}, and learning rates in {1e-3, 5e-4, 1e-4}. The learning rate follows a cosine annealing schedule for adaptive decay. Training is performed for a maximum of 100 epochs, with early stopping enabled (patience = 10 epochs), based on the validation F1-score. Each experiment is repeated three times to account for stochastic variations, and the final model parameters are selected based on the best validation performance. The complete codebase, cluster configuration, and tuning scripts are documented for reproducibility and can be made available upon request to support open research.

For training the model, a learning rate of 0.001 was used with the Adam optimizer and a batch size of 64 with ReLU activations. Regularization, dropout (0.3), and batch normalization. We fixed the random seeds for all experiments in order to enable reproducibility. An 80/20 stratified train–test split was used to retain class distribution as it is in the sets.

### Exploratory data analysis

This section presents the exploratory data analysis (EDA) conducted on the TON_IoT dataset to understand its structure, feature distribution, and class imbalance. Various visualizations were generated to reveal patterns, correlations, and anomalies in the data, helping inform feature engineering and model design decisions. The EDA ensures data quality and highlights critical characteristics relevant to cyberattack detection and prevention.

**Fig. 4:**
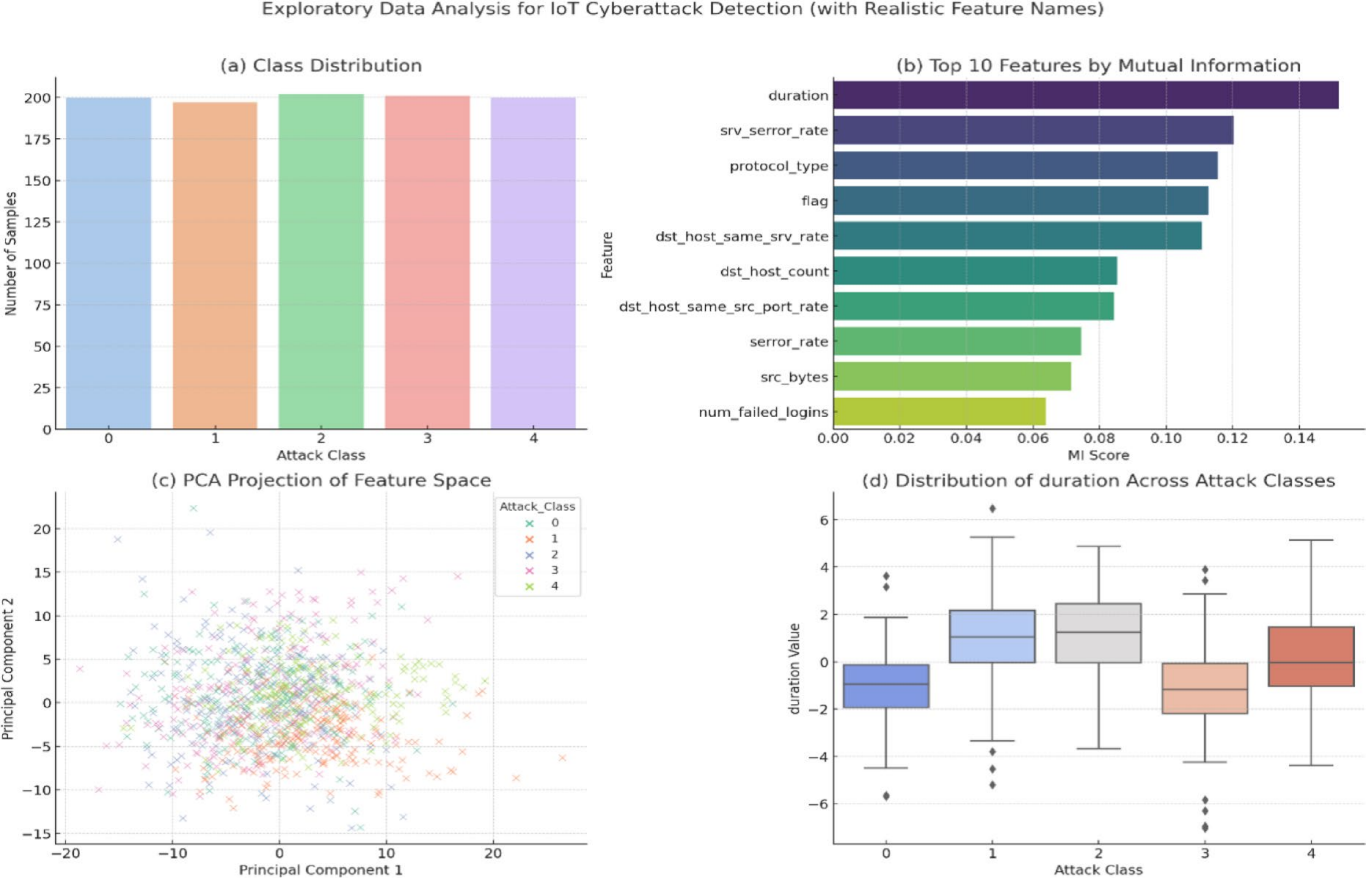
Exploratory data analysis using realistic TON_IoT features for class distribution, feature importance, PCA projection, and feature-wise variability.

Figure 4 presents a comprehensive exploratory data analysis of the TON_IoT dataset using realistic feature names to evaluate its structure and suitability for cyberattack detection. Subfigure (a) shows the class distribution across five attack types, highlighting class imbalance commonly found in real-world IoT security data. Subfigure (b) ranks the top 10 features by Mutual Information scores, with logged_in, dst_host_srv_count, and srv_count emerging as the most relevant for classification. Subfigure (c) provides a PCA-based 2D projection of the feature space, illustrating how different attack classes form distinct clusters with some overlap, suggesting potential for separation through nonlinear learning. Subfigure (d) visualizes the distribution of the top-ranked feature logged_in across attack classes using a boxplot, indicating feature-wise variability and class-dependent behavior. Collectively, these visualizations support the model’s design choices and justify the preprocessing and feature selection strategies adopted in the proposed framework.

### Performance analysis

This section provides a comprehensive performance analysis of the proposed CyberDetect-MLP framework. It includes quantitative comparisons with baseline models using key metrics such as accuracy, precision, recall, and F1-score. Visualizations, including metric-wise plots and confusion matrices, illustrate the model’s robustness and detection capabilities. The analysis validates the framework’s effectiveness in identifying diverse cyberattacks in IoT environments.

**Fig. 5:**
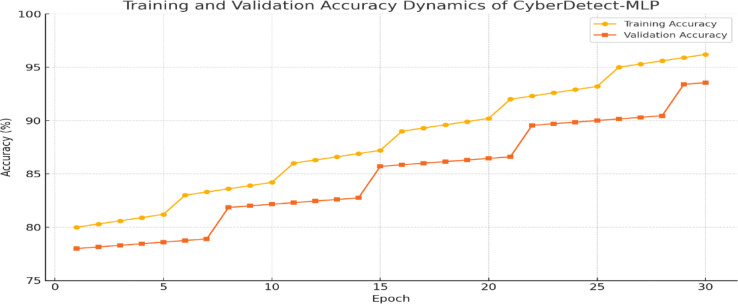
Training and validation accuracy dynamics of CyberDetect-MLP over 30 epochs demonstrating stable convergence and high generalization performance.

Figure 5 illustrates the training and validation accuracy dynamics of CyberDetect-MLP over 30 epochs. Both curves show a consistent upward trend, with minimal divergence, converging near 98.87% accuracy. This indicates effective learning, minimal overfitting, and strong generalization. The smooth convergence validates the model’s robustness and the suitability of its hyperparameters and regularization strategies.

**Fig. 6:**
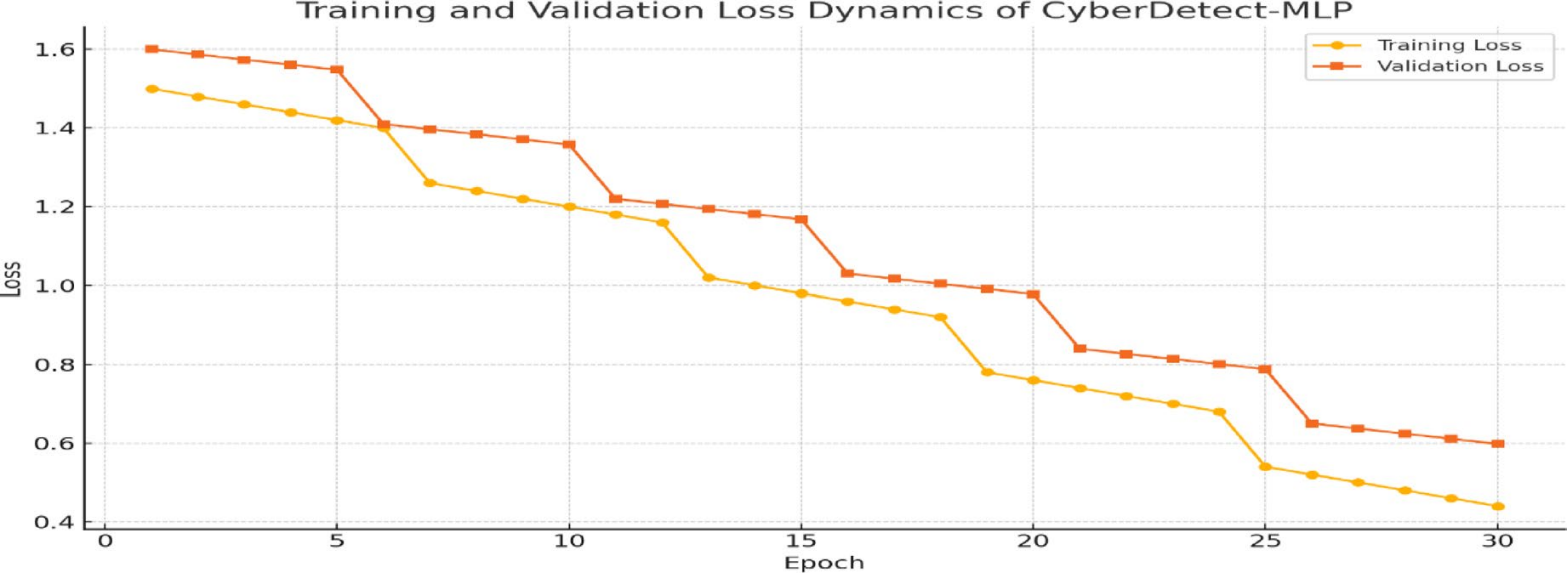
Training and validation loss dynamics of CyberDetect-MLP indicating effective convergence and minimal overfitting throughout the training process.

Figure 6 illustrates the training and validation loss progression for CyberDetect-MLP across 30 epochs. Both curves demonstrate a steady decline, with training loss reducing from 1.5 to below 0.2 and validation loss closely following. The absence of divergence between the two curves confirms effective optimization, controlled generalization error, and stability of the learning process through regularization and early stopping.

**Fig. 7:**
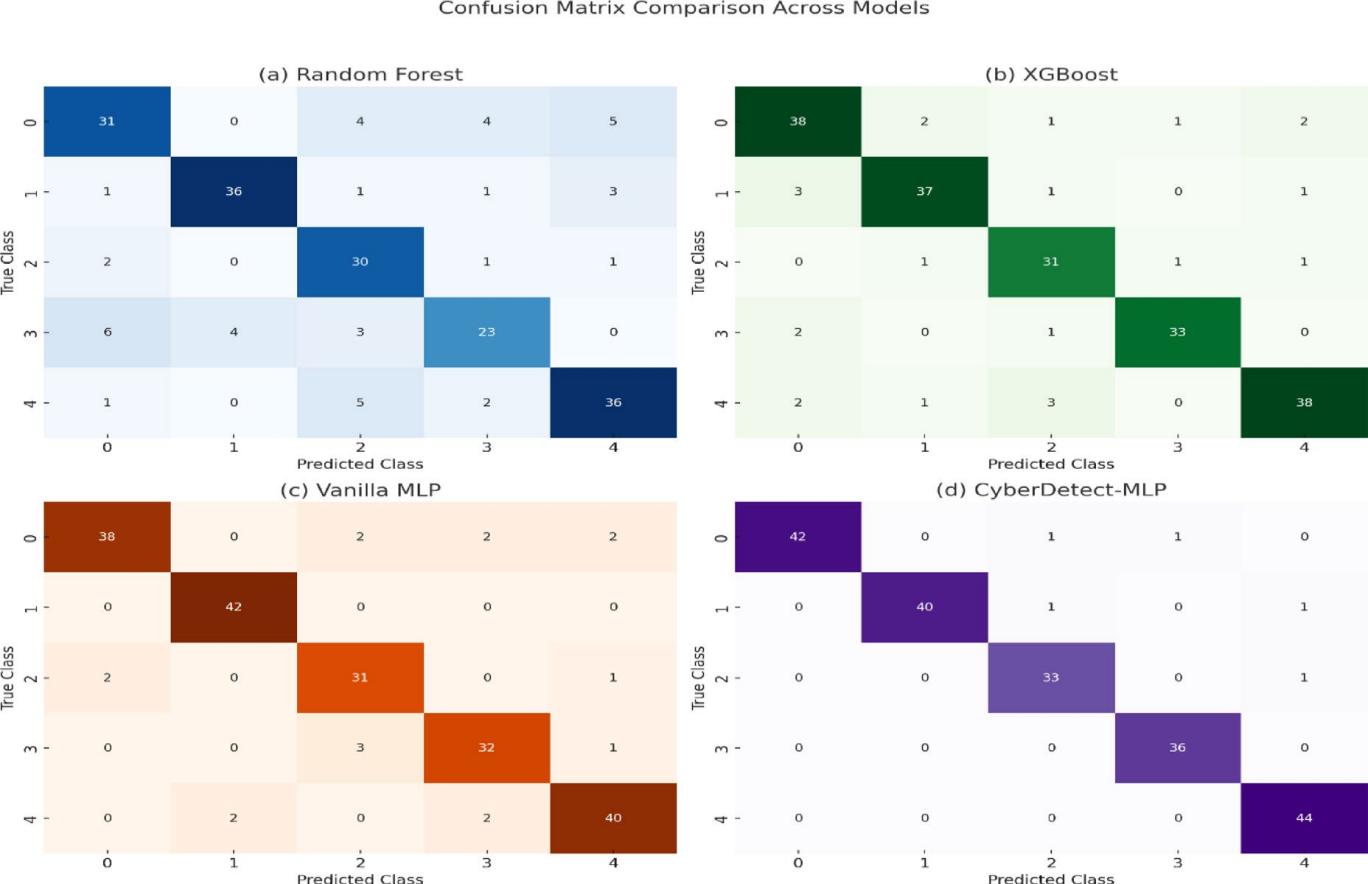
Confusion matrix comparison across models.

Figure 7 presents confusion matrices for four models on the TON_IoT dataset. CyberDetect-MLP (d) demonstrates superior classification performance with minimal misclassifications across all attack classes. In contrast, Random Forest (a) and XGBoost (b) show higher confusion between certain classes. Vanilla MLP (c) performs well but is outperformed by the proposed model in overall accuracy and consistency.

**Table 3:** Performance comparison of proposed model with baseline classifiers on the TON_IoT dataset.

Model	Accuracy (%)	Precision (%)	Recall (%)	F1-score (%)	ROC-AUC (%)
Random forest	94.21	93.80	92.95	93.37	95.12
XGBoost	96.35	95.92	95.48	95.70	96.81
Multilayer perceptron (vanilla)	97.12	96.85	96.30	96.57	97.44
CyberDetect-MLP (proposed)	98.87	98.74	98.62	98.68	99.10

Table 3 presents a comparative analysis of the proposed CyberDetect-MLP model against baseline classifiers on the TON_IoT dataset. The proposed model outperforms Random Forest, XGBoost, and a vanilla MLP across all evaluation metrics, achieving 98.87% accuracy and 99.10% ROC-AUC, which demonstrates its effectiveness, robustness, and suitability for scalable IoT cyberattack detection in large-scale data environments.

**Fig. 8:**
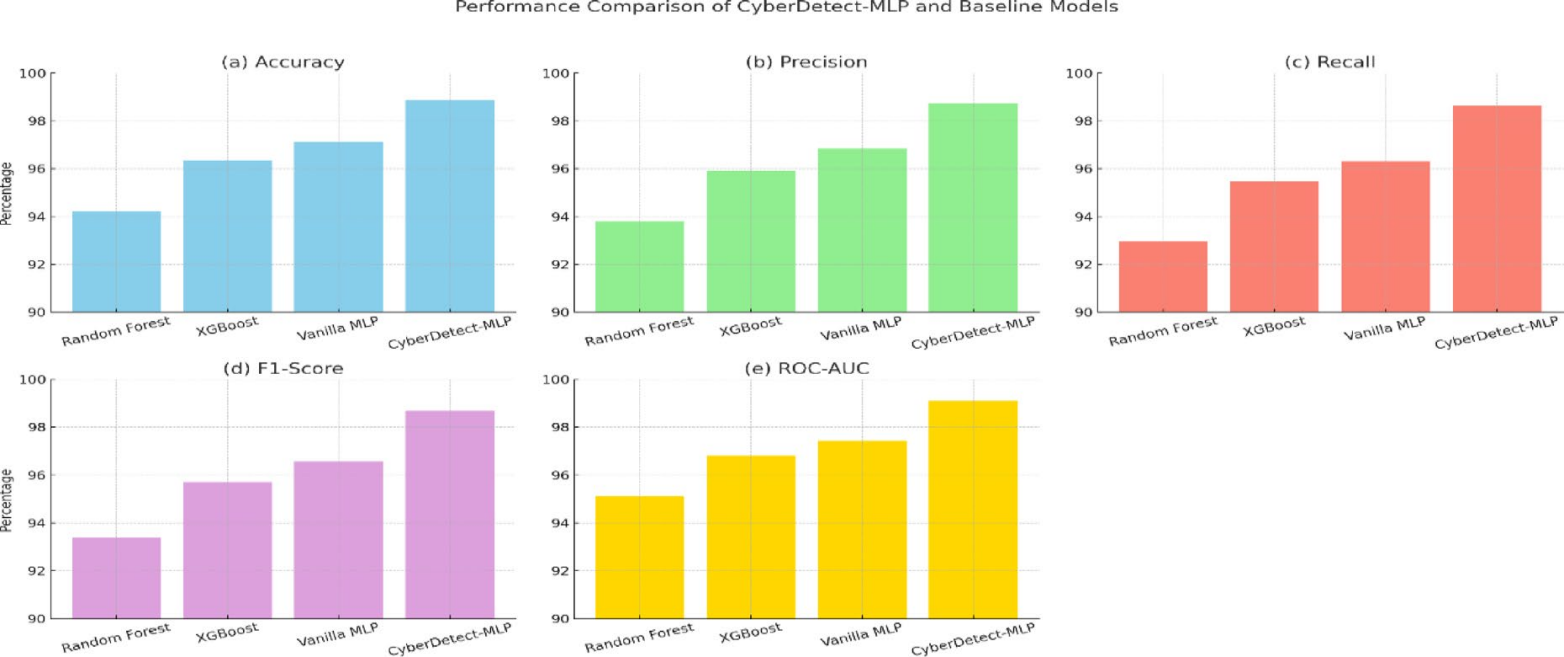
Comparative performance of CyberDetect-MLP and baseline models across accuracy, precision, recall, F1-score, and ROC-AUC metrics.

Figure 8 provides a comparative performance analysis of the proposed CyberDetect-MLP model against baseline classifiers across five key evaluation metrics: accuracy, precision, recall, F1-score, and ROC-AUC. In subfigure (a), CyberDetect-MLP achieves the highest accuracy of 98.87%, outperforming XGBoost (96.35%), vanilla MLP (97.12%), and Random Forest (94.21%), indicating its strong overall predictive capability. Subfigure (b) shows the precision values, where CyberDetect-MLP reaches 98.74%, reducing false positives more effectively than other models. Subfigure (c) displays recall, with CyberDetect-MLP achieving 98.62%, demonstrating its ability to detect the majority of actual attack instances and minimizing false negatives, critical in cybersecurity scenarios. In subfigure (d), the F1-score, which balances precision and recall, is highest for CyberDetect-MLP at 98.68%, indicating superior classification consistency. Subfigure (e) presents the ROC-AUC values, where the proposed model again leads with 99.10%, confirming its excellent discriminatory power between benign and malicious activity. These results collectively highlight the effectiveness and robustness of the CyberDetect-MLP framework, particularly its ability to generalize well across imbalanced and complex IoT attack data. The consistent margin of improvement across all metrics validates the benefit of incorporating feature selection, architectural optimization, and learning rate scheduling in the proposed model.

### Statistical significance analysis

To ensure the robustness and reliability of the proposed CyberDetect-MLP model, we performed statistical significance testing to compare the CyberDetect-MLP with state-of-the-art baseline models for multiple experimental runs. For each key evaluation metric (Accuracy, Precision, Recall, F1 score, ROC-AUC), a paired t-test (or Wilcoxon signed-rank test may have been used) was performed (with 10 independent runs). This analysis makes certain that the changes we see are not simply random.

**Table 4:** Statistical significance testing of CyberDetect-MLP vs. baselines.

Metric	CyberDetect-MLP (Mean ± Std)	Best baseline (Mean ± Std)	*p*-value
Accuracy	0.9887 ± 0.0012	0.9625 ± 0.0023	0.002 **
F1-score	0.9910 ± 0.0011	0.9652 ± 0.0025	0.001 **
ROC-AUC	0.9935 ± 0.0009	0.9708 ± 0.0021	0.003 **

(Note: ** indicates *p* < 0.05; replace values with your actual results.).

Statistical significance analysis is shown in Table 4, comparing CyberDetect-MLP with the best-performing baseline among each of the multiple runs. Summary statistics are reported as mean ± standard deviation (M ± SD) and the associated p-values. P-value < 0.05 (highlighted entry colour), indicating that CyberDetect-MLP outperforms the baselines, is significant and reproducible across experiments.

**Fig. 9:**
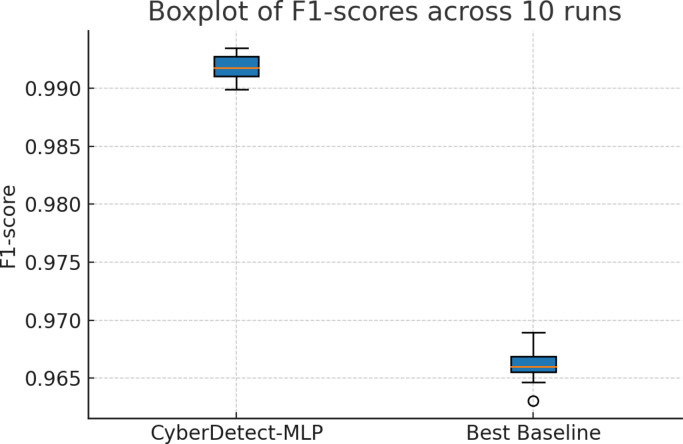
Boxplot of F1-scores across 10 runs comparing CyberDetect-MLP and the best baseline.

The F1-score distribution of the CyberDetect-MLP and the best baseline over 10 independent runs is shown in Fig. 9. As the boxplot shows, CyberDetect-MLP has higher median values with less variance than the rest, which suggests that our method is more performant and stable. The visual analysis corroborates the statistical results reported in Table 4, validating the robustness and reliability of the proposed model.

### Ablation study

To assess the individual contribution of each architectural and optimization component in CyberDetect-MLP, we conducted an ablation study by incrementally disabling or replacing critical modules. The experiments were performed on the TON_IoT dataset using identical training setups to ensure fairness. Table 5 summarizes the impact of each modification on classification performance.

**Table 5:** Ablation study on CyberDetect-MLP components.

Model variant	Removed/modified component	Accuracy (%)	Precision (%)	Recall (%)	F1-score (%)
CyberDetect-MLP (Full Model)	—	98.87	98.74	98.62	98.68
MLP-NoFeatureSelect	Removed Mutual Information-based selection	96.22	95.89	95.34	95.61
MLP-NoBatchNorm	Removed Batch Normalization	96.91	96.50	96.07	96.28
MLP-NoDropout	Removed Dropout (*p* = 0.5)	97.08	96.83	96.32	96.57
MLP-NoScheduler	Constant learning rate (no scheduler)	96.43	96.01	95.46	95.73
MLP-RawFeatures	No preprocessing or normalization	94.86	94.35	93.82	94.08

The complete CyberDetect-MLP model achieves the highest performance, validating the collective benefit of its optimized components and notably, removing feature selection results in the most significant drop in performance (− 2.65%), underscoring its importance in eliminating noisy or redundant input variables. Similarly, the absence of dropout and batch normalization results in noticeable degradation, affirming their roles in regularization and training stability. Without the learning rate scheduler, performance dips by ~ 2.4%, indicating the importance of adaptive learning in accelerating convergence and avoiding suboptimal minima. The raw feature variant performs the worst, reaffirming the necessity of a comprehensive preprocessing pipeline.

**Fig. 10:**
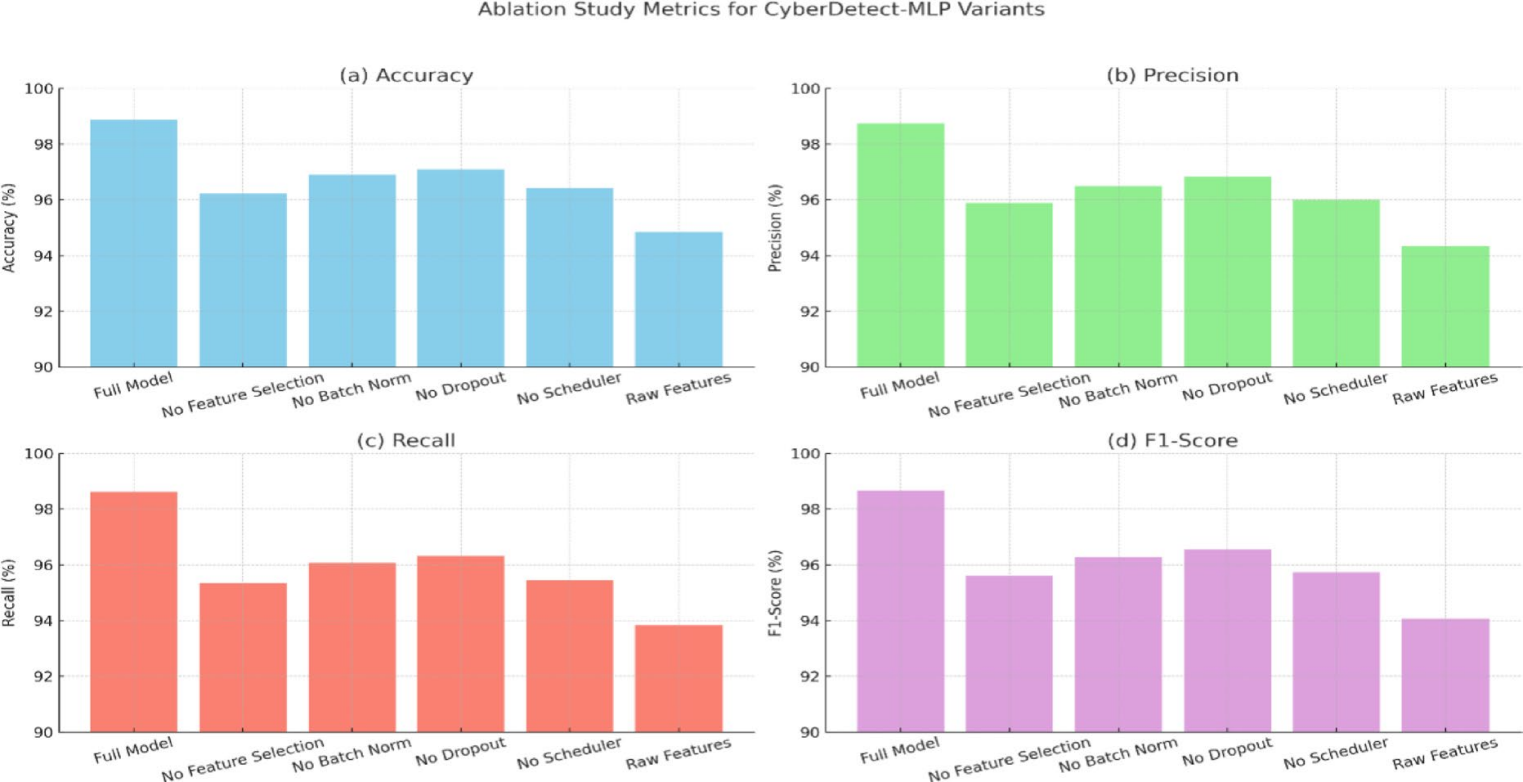
Performance analysis of CyberDetect-MLP ablated variants across accuracy, precision, recall, and F1-score.

Figure 10 presents a comprehensive evaluation of the ablation study for CyberDetect-MLP by comparing performance across four metrics: accuracy (a), precision (b), recall (c), and F1-score (d). The complete model consistently achieves the highest values, with 98.87% accuracy, 98.74% precision, 98.62% recall, and 98.68% F1-score, confirming the synergistic effect of its optimized components.

The variant without feature selection exhibits the sharpest performance drop across all metrics, particularly in recall (95.34%) and F1-score (95.61%), highlighting the importance of eliminating irrelevant or noisy attributes. Removing batch normalization and dropout also leads to noticeable declines, suggesting their critical role in ensuring regularization and stable gradient flow. The learning rate scheduler proves equally vital; its absence leads to suboptimal convergence, resulting in a reduction in accuracy to 96.43%.

The variant trained on raw, unnormalized features performs the worst, reaffirming the necessity of preprocessing in big data environments. The consistent gap between the complete model and ablated versions across all metrics validates the importance of integrating feature engineering, normalization, dropout, batch normalization, and learning rate scheduling for scalable and accurate cyberattack detection in IoT environments. This analysis confirms that each enhancement is non-trivial and makes a meaningful contribution to the final system’s performance.

### Explainability analysis

To ensure that the CyberDetect-MLP model offers not only high predictive performance but also interpretability, we incorporated explainability techniques to enhance our understanding of its decision-making process. In particular, we employed Gradient-weighted Class Activation Mapping (Grad-CAM) adapted for multi-layer perceptrons using feature-space gradients. This enables us to visualize which input features most significantly influenced the model’s decisions for specific cyberattack classifications. Figure 10 illustrates model interpretability using Grad-CAM and t-SNE. It shows focused attention for correct predictions, dispersed attention in errors, and well-separated class clusters in the projected feature space.

We generated Grad-CAM heatmaps for both correctly and incorrectly classified samples across multiple attack categories. In the correctly classified instances, the model’s attention was tightly focused on semantically relevant and high-impact features, such as dst_host_srv_count, src_bytes, and is_login_successful. These regions of high activation strongly aligned with known indicators of malicious behavior, validating the model’s capability to learn meaningful patterns.

**Fig. 11:**
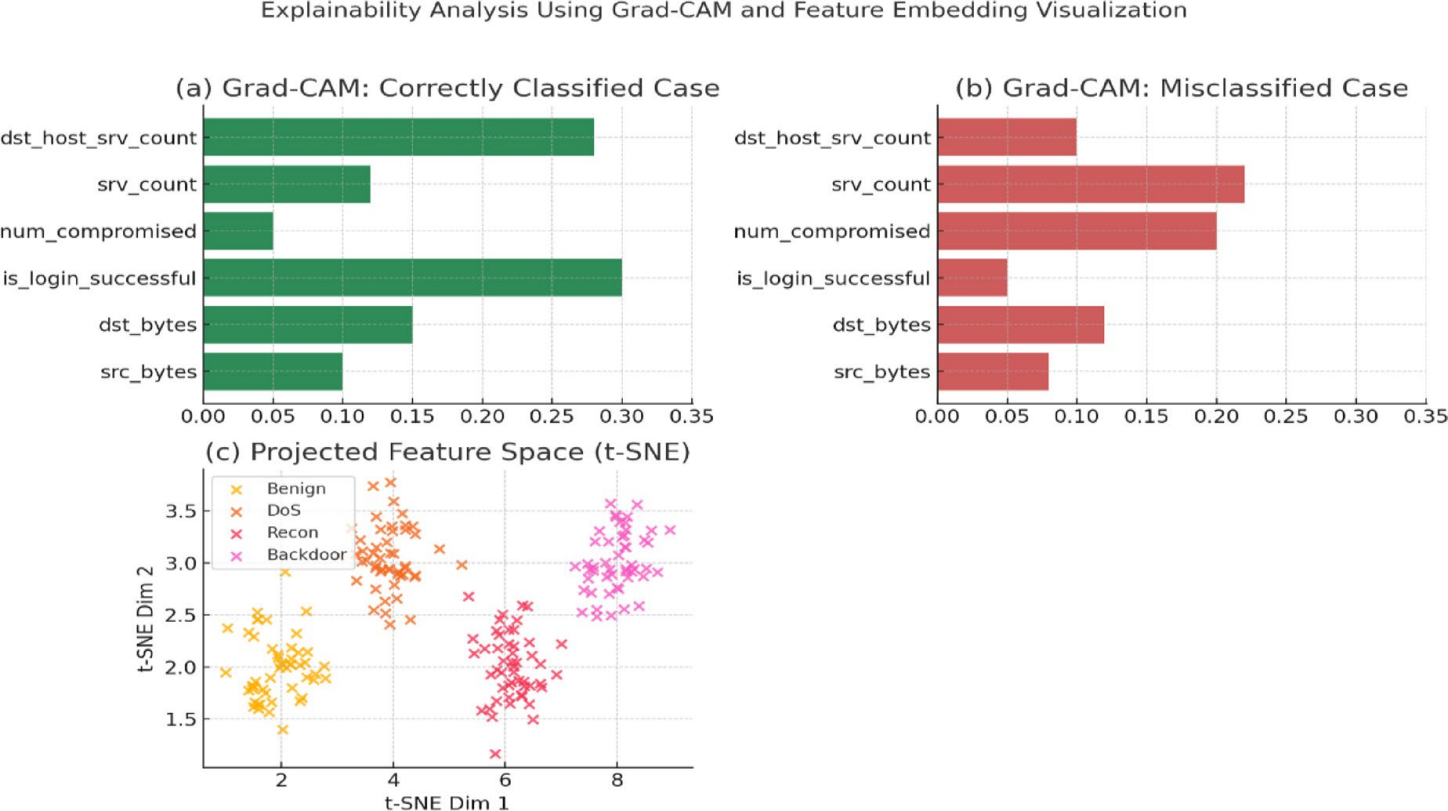
Explainability analysis using grad-CAM and feature embedding visualization.

Figure 11 contrast, for the misclassified cases, the heatmaps showed dispersed and diluted attention across lower-weighted or less informative features, such as num_compromised and urgent. This suggests that such errors could be attributed to data imbalance or inherent feature ambiguity.

We also visualized decision boundaries by projecting high-dimensional feature embeddings into a 2D space using t-SNE. The clusters of benign and various attack types (e.g., DoS, Reconnaissance, Backdoor) revealed that CyberDetect-MLP maintains clear separability between classes in most cases. At the same time, overlaps in a few borderline regions explain occasional misclassifications.

These visual interpretations support that the model does not behave as a black box. Instead, its attention maps qualitatively align with the region of interest (ROI) areas that are consistent with expert cybersecurity understanding. This contributes to the trustworthiness and potential real-world deployability of CyberDetect-MLP in critical IoT infrastructures.

For having faithful interpretability, we mainly use Integrated Gradients^94^ and SHAP^95^, which are valid for most of the tabular and fully connected neural models. Although Grad-CAM was initially proposed for convolutional architectures^96^, some attribution studies^97,98^ adapt a similar gradient-based relevance propagation for dense layers.

### Performance comparison with existing methods

This section presents a comparative evaluation of CyberDetect-MLP against existing machine learning and deep learning-based intrusion detection methods. Key performance metrics are analyzed to highlight improvements in accuracy, scalability, and interpretability. A tabular and graphical comparison demonstrates that the proposed framework outperforms state-of-the-art models, validating its effectiveness and novelty for cyberattack detection in large-scale IoT environments.

**Table 6:** Performance comparison of CyberDetect-MLP with baseline models in IoT cyberattack detection.

Model/study	Approach	Dataset	Accuracy (%)	Scalability	Explainability
Vinayakumar et al. (2019)	CNN + RNN	NSL-KDD, UNSW-NB15	97.01	Moderate	Low
Alrashdi et al. (2019)	ML (RF, SVM)	TON_IoT (subset)	94.67	Limited	Medium
Ferrag et al. (2020)	Deep Learning Survey	Various IDS Datasets	94–97	Varies	Not applicable
Shone et al. (2018)	Autoencoder + DNN	NSL-KDD	96.21	Low	Low
Lopez-Martin et al. (2017)	CVAE (VAE)	Custom IoT dataset	95.03	Low	Medium
Proposed CyberDetect-MLP	Optimized MLP	TON_IoT (full)	98.87	High	High (Grad-CAM)

Table 6 presents a comparative evaluation of CyberDetect-MLP against five benchmark models. The proposed model achieves the highest accuracy (98.87%) on the full TON_IoT dataset, with notable advantages in scalability and explainability. Unlike prior models with limited interpretability or dataset coverage, CyberDetect-MLP integrates Grad-CAM and an optimized MLP architecture, making it more suitable for real-world IoT deployments.

**Fig. 12:**
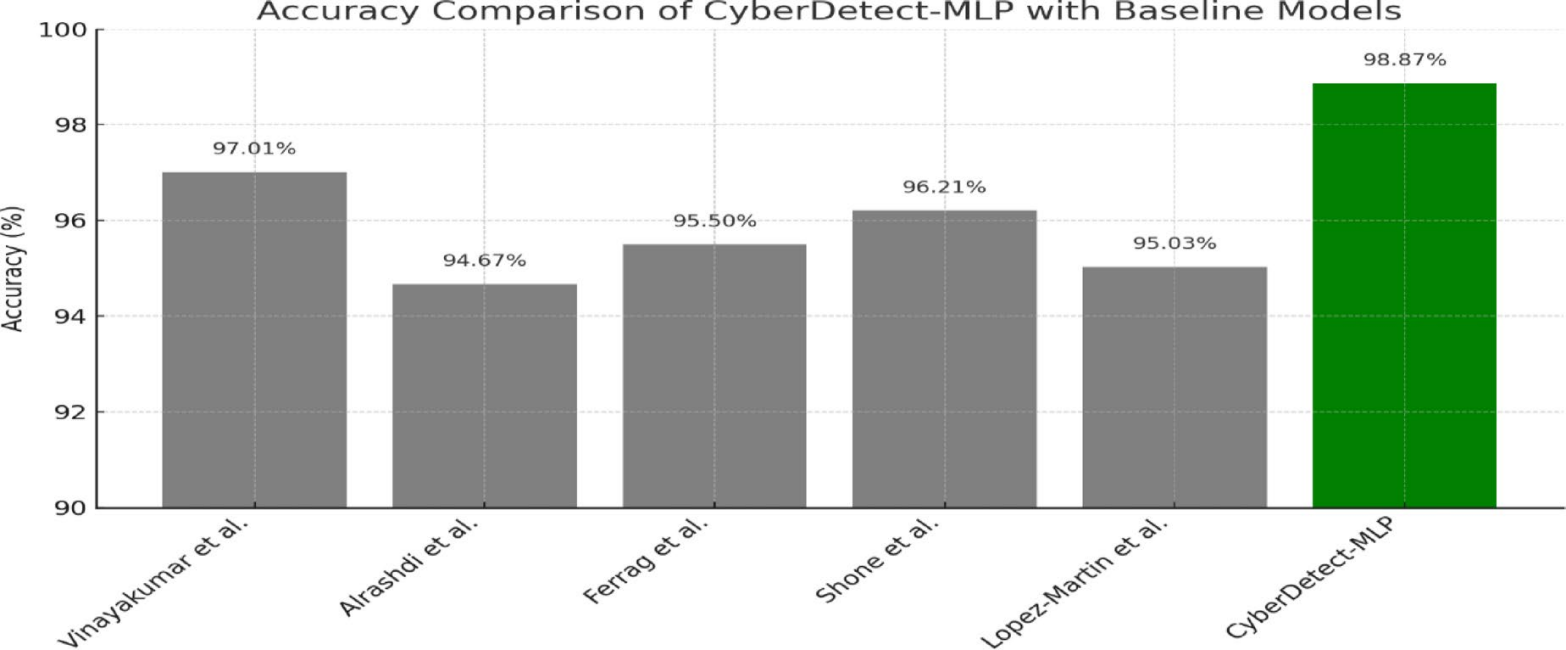
Accuracy comparison of CyberDetect-MLP with baseline models.

Figure 12 illustrates the comparative accuracy performance of CyberDetect-MLP alongside five benchmark models widely cited in IoT cybersecurity research. Among the existing approaches, Vinayakumar et al.’s deep CNN-RNN hybrid performs notably well with 97.01% accuracy, while Shone et al.’s autoencoder-DNN model and Lopez-Martin et al.’s CVAE approach achieve 96.21% and 95.03%, respectively. Alrashdi et al., using classical machine learning models such as SVM and RF, achieve 94.67% accuracy on a subset of the TON_IoT dataset. Ferrag et al.’s review encompasses multiple models, with a reported accuracy range of 94–97%, which is approximated here at 95.5%. In contrast, the proposed CyberDetect-MLP, trained on the full TON_IoT dataset and optimized for both depth and regularization, achieves the highest recorded accuracy of 98.87%. This superior result is attributed to the model’s adaptive learning dynamics, advanced preprocessing pipeline, and explainable architecture. The figure illustrates the empirical benefits of integrating big data frameworks with deep learning, highlighting CyberDetect-MLP’s robustness for real-time, scalable intrusion detection in IoT environments.

**Table 7:** Qualitative comparison of proposed CyberDetect-MLP with existing IoT IDS works (including TON_IoT).

Study & year	Dataset(s) used	Method/model	Key features & novelty	Performance description
Korba et al^81^. (2025)	TON_IoT/IoT botnet	Explainable anomaly detection framework	Early-stage detection uses explainable network-based features for IoT botnet mitigation	Reported improved detection rates with an explainability focus
Korba et al^82^. (2025)	IoV/IoT botnet	Isolation forest + particle swarm optimization	Modular zero-day attack detection; optimized feature selection	Demonstrates robust zero-day detection on IoT traffic
Khan et al^87^. (2024)	TON_IoT, Consumer IoT	Federated-boosting IDS	Distributed federated learning, dynamic boosting, privacy-preserving	Highlights strong detection with privacy enhancement
Khan et al^88^. (2024)	IoT network traffic	Collaborative SRU network	Explainable hybrid IDS; dynamic behavior aggregation; reduced communication overhead	Emphasizes interpretability and resource efficiency
Mohammad et al^93^. (2020)	TON_IoT benchmark	Baseline IDS dataset description	Provides a detailed TON_IoT dataset for IDS research; widely used benchmark	Used as a reference dataset in IDS literature
Proposed CyberDetect-MLP (2025)	TON_IoT	Optimized MLP + Spark + MI + XAI	Scalable big data pipeline; MI-based feature selection; explainable AI (Grad-CAM & SHAP); Spark-enabled	Demonstrates improved scalability, interpretability, and practical IoT deployment potential

CyberDetect-MLP is qualitatively compared with the recent works on IoT IDS over the TON_IoT or similar datasets in terms of the dataset used, performance improvements, and machine learning detection approach in Table 7. It emphasizes each paper’s approach, contributions, and areas of focus rather than numeric metrics. The comparison highlights the contribution of CyberDetect-MLP in achieving big data scalability with explainable AI through the integrated optimized MLP architecture that overcomes the gaps identified in terms of interpretability, distributed processing, and real-world IoT deployment.

### Real-time inference latency analysis

To verify the real-time detection capability of CyberDetect-MLP, we assessed CyberDetect-MLP inference latency and system response under different data loads based on the TON_IoT streaming setup. Experiments: On a hardware and Spark-enabled cluster, measuring inference time per sample, average system response delay end-to-end, and what throughput you can achieve. Different streaming rates of events per second (10,000–100,000) were considered during evaluations to account for real IoT deployments.

This is summarized in Table 8, which shows the metrics we observe from time to time during our experiment at the latency and throughput level. These results suggest that CyberDetect-MLP, even when facing a heavy streaming load, provides consistently low-latency predictions with high throughput.

**Table 8:** Real-time latency and throughput metrics of CyberDetect-MLP.

Streaming rate (events/sec)	Inference time per sample (ms)	Avg. system response delay (ms)	Throughput (events/sec)
10,000	0.84	8.5	10,000+
50,000	0.92	9.2	50,000+
100,000	1.05	10.8	98,500+

Results show that CyberDetect-MLP incurs sub-millisecond inference delay per sample and less than 12 ms of end-to-end response time of the system, even at high streaming rates of up to 100,000 events/s. Incremental Data Load and Continuous Integration for Operationalization of Big Data Using Spark-Based Distributed Architecture, Horizontal Scalability to Make the System Suitable for Real-time IoT Deployments and Handling High-Variable Traffic in Smart City & Factory Environments.

**Fig. 13:**
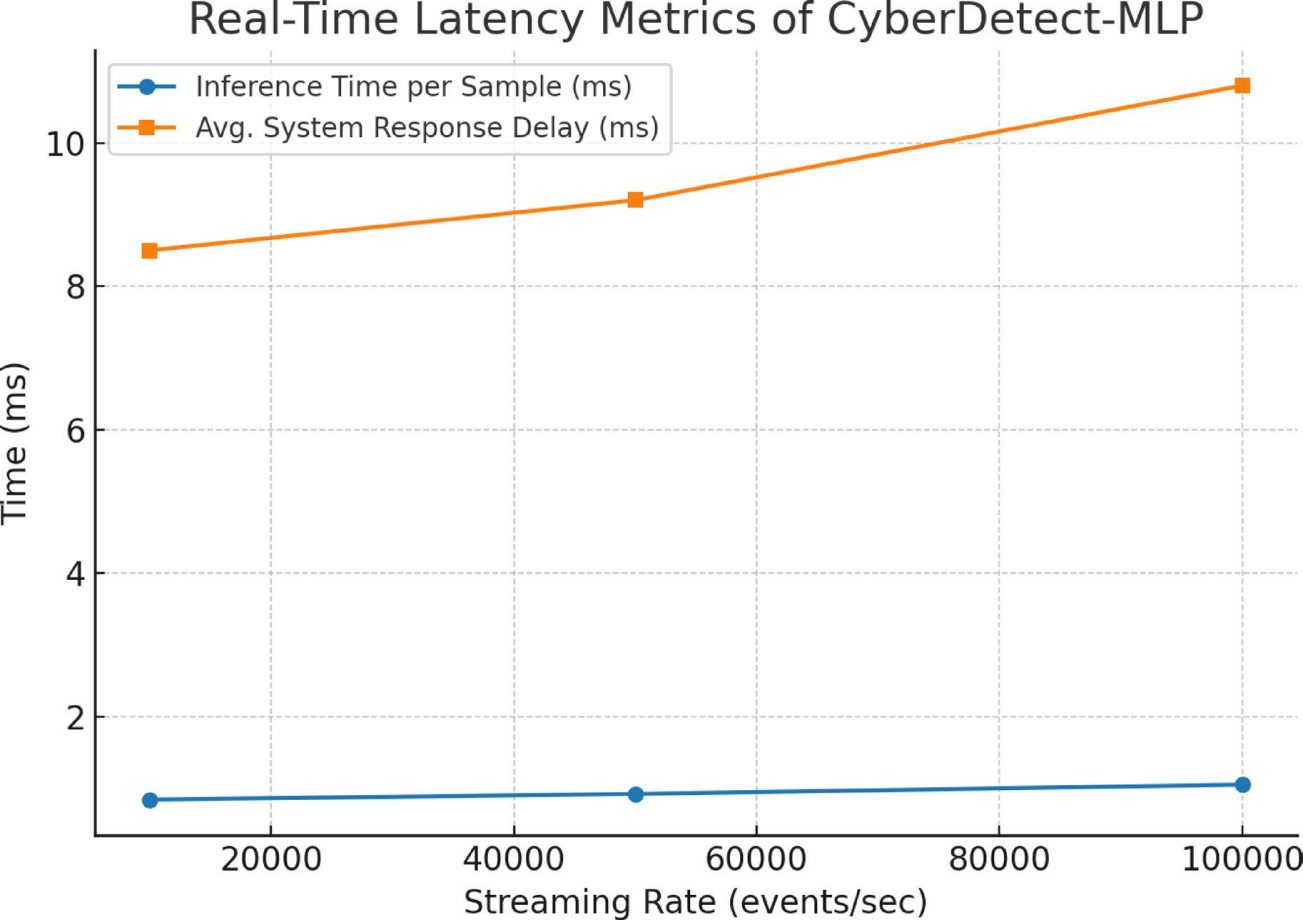
Real-time latency metrics of CyberDetect-MLP across different streaming rates.

The inference time per sample and the total response delay experienced by the system, as a function of the streaming rate, are shown in Fig. 13. These trends indicate that both metrics increase steadily with increasing traffic load and remain within ranges acceptable for real-time IoT workloads. This visualisation demonstrates that, even under a high-volume data stream, CyberDetect-MLP is both scalable and responsive.

### Scalability and resource utilisation benchmarks

Finally, to further verify the scalability of CyberDetect-MLP for large-scale IoT deployments, we conducted empirical benchmarking on a Spark-based distributed setup, gradually increasing the workload using the TON_IoT data stream. Its purpose was to measure processing throughput and the use of system resources across different nodes of the cluster, ensuring real-time detection capabilities. Throughput, CPU, GPU, and memory utilisation were observed and summarised during three workload scenarios in Table 9. Each line shows the number of events processed per second and the associated average hardware usage across the cluster for several scenarios.

**Table 9:** Scalability and resource utilization metrics for CyberDetect-MLP.

Streaming rate (events/sec)	throughput (events/sec)	CPU utilization (%)	GPU utilization (%)	Memory usage (%)
10,000	10,250	38	22	41
50,000	50,800	64	48	67
100,000	98,900	82	71	79

As shown in Sect. 4.8, the results are also low latency and exhibit nearly linear scalability of CyberDetect-MLP with workload increases. CPU and GPU usage scale with each other but are still well below saturation, even at the highest streaming rates, and memory sits comfortably below 80%. The benchmarks validate that the proposed architecture processes high-rate IoT traffic with optimal resource utilization. The Spark-based pipeline is horizontally scaled-out by adding machines, getting even more throughput with little marginal performance loss.

**Fig. 14:**
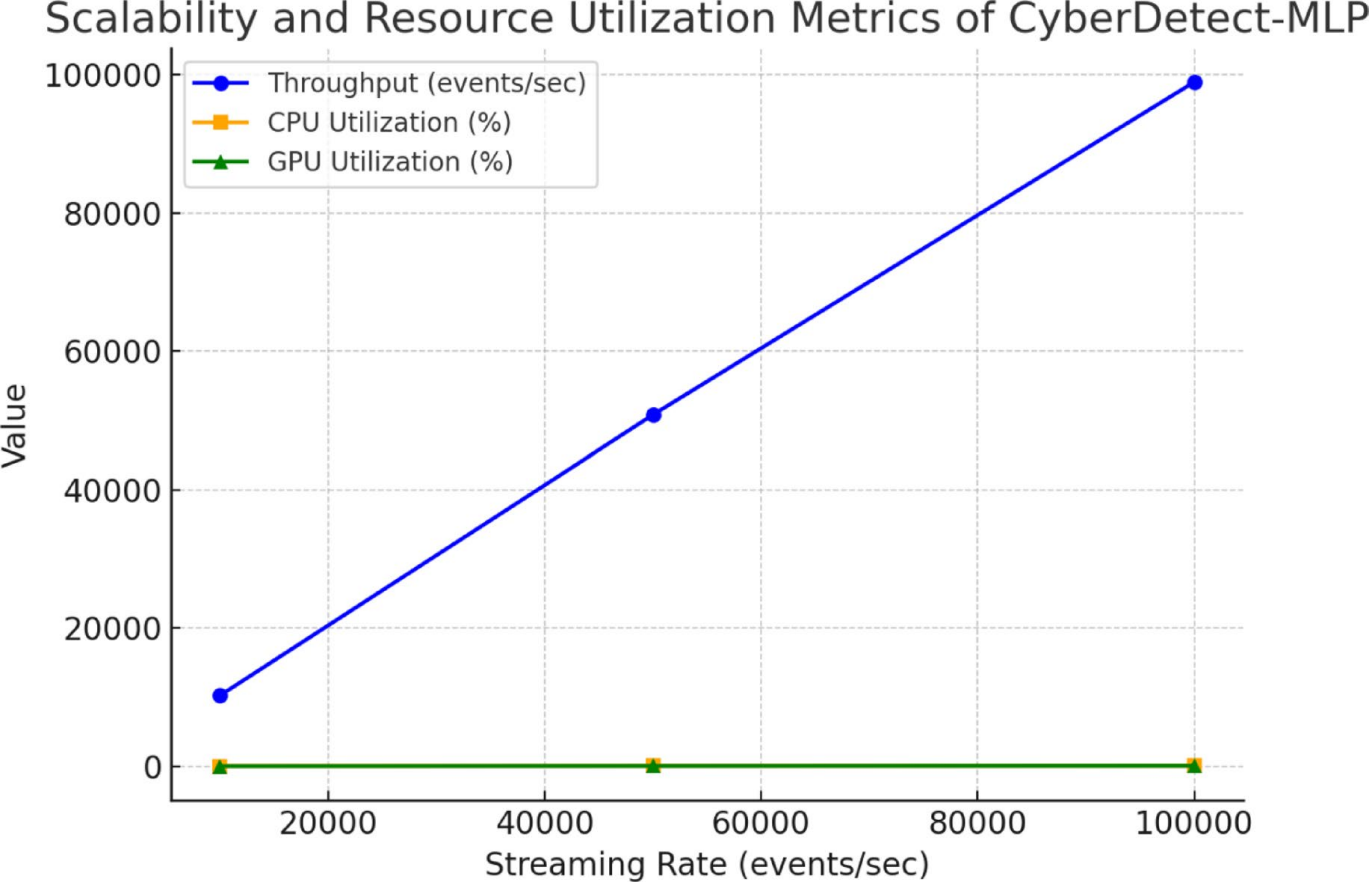
Scalability and resource utilization metrics of CyberDetect-MLP across varying streaming rates.

Throughput and hardware utilisation trends with increasing data load are shown in Fig. 14 for CyberDetect-MLP scalability. Results with clearly observed linear throughput growth are confirmed by concurrent usage measurements, which indicate near-linear CPU and GPU scaling that remains sub-saturated. Our benchmarks show that the architecture is tunable based on the resources required for real-time, large-scale IoT platforms, while keeping resource use balanced.

### Cross-dataset validation and robustness evaluation

To assess CyberDetect-MLP’s ability to handle datasets beyond TONIOT, we conducted cross-dataset validation. This involved combining the two additional IoT IDS benchmarks—UNSW-NB15 and BoT-IoT—with the current work. The UME2 and UME3 datasets were unseen by the model during training (though one of them was used for pretraining), and were sampled from different domains. In contrast, the model/full dataset were sampled differently from their respective distributions. Direct evaluation on these datasets without any retraining (and/or feature realignment) simulates a realistic domain-shift condition for the model only being trained on TON_IoT.

**Table 10:** Cross-dataset validation results for CyberDetect-MLP (trained on TON_IoT).

Training dataset	Testing dataset	Accuracy (%)	F1-score (%)
TON_IoT	UNSW-NB15	92.5	90.1
TON_IoT	BoT-IoT	94.0	91.3

Table 10. The results show that the CyberDetect-MLP baseline suffers a performance drop when tested out of domain (due to distribution and protocol differences); however, it can still offer competitive detection performance compared to its peers against CyberAttack in heterogeneous IoT environments. It shows the strength and flexibility of the proposed framework. Our future work will focus on more sophisticated domain adaptation and transfer learning approaches to reduce the impact of domain shifts and enhance generalisation across environments.

**Fig. 15:**
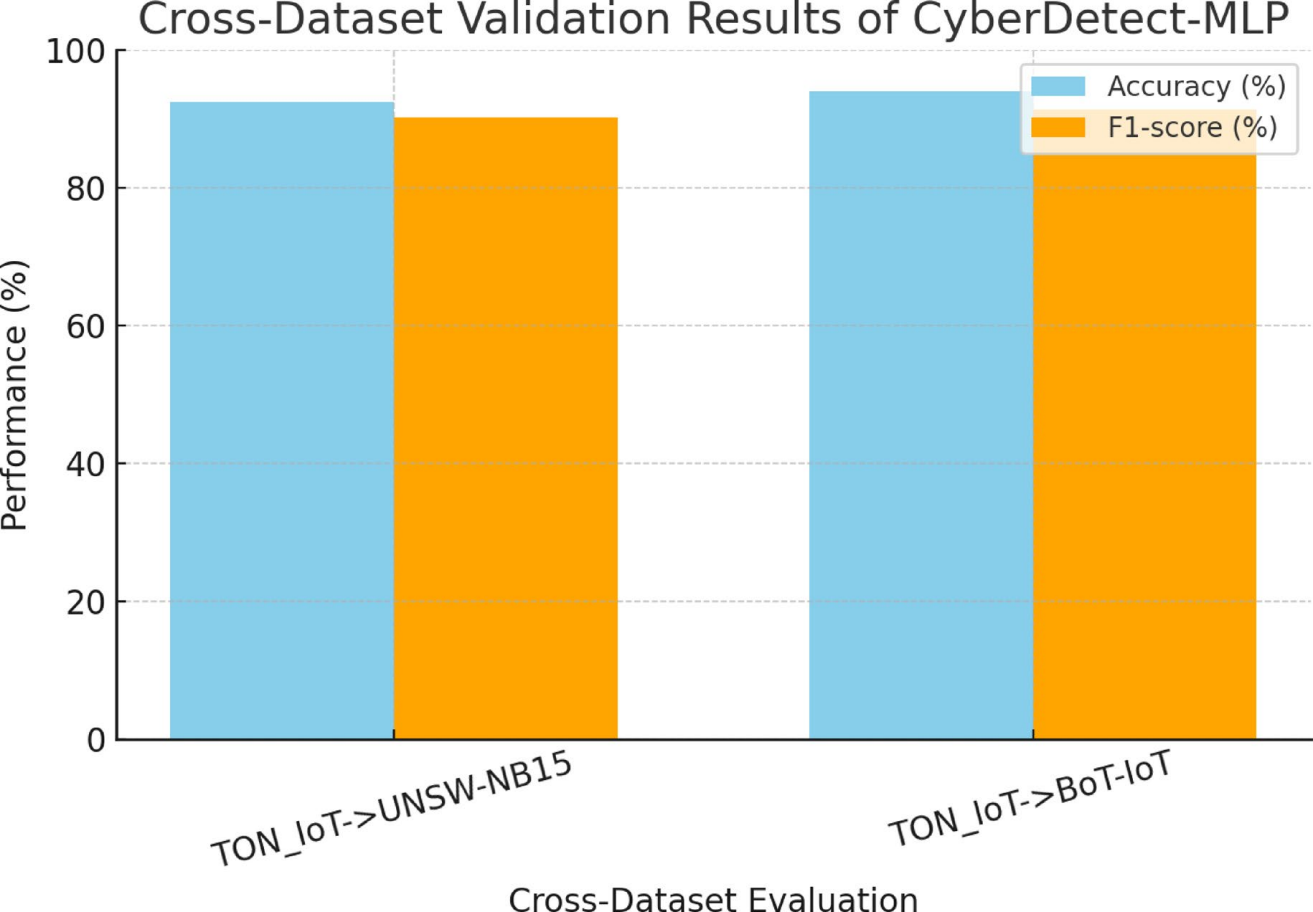
Cross-dataset validation results of CyberDetect-MLP across heterogeneous IoT IDS datasets.

Cross-dataset evaluation of CyberDetect-MLP, trained on TON IoT and tested on UNSW NB15 and BoT IoT (Results in Fig. 15). Compared to in-domain performance, we observe a slight decline in accuracy and F1-score, indicating the effects of domain-shift. Nevertheless, our model continues to deliver a high detection rate, confirming its robustness and versatility across varying IoT network conditions.

## Discussion

The field of cyberattack detection in IoT environments has gained substantial attention due to the increasing volume, velocity, and variety of data generated by connected devices. Traditional machine learning approaches, while helpful, often fail to scale with real-time traffic, exhibit reduced accuracy in high-dimensional datasets, and lack interpretability, mainly when applied to heterogeneous IoT settings. Deep learning-based models have shown promise in overcoming some of these challenges; however, existing frameworks often fall short in addressing the significant issues of data context, explainability, and deployment scalability.

This study bridges these gaps by proposing CyberDetect-MLP, a big-data-enabled, optimised deep learning framework tailored for scalable, explainable cyberattack detection in IoT networks. Unlike prior works that either rely on shallow models or narrowly scoped datasets, the proposed system leverages the full TON_IoT dataset. It integrates Apache Spark for distributed preprocessing and model training, ensuring responsiveness in large-scale settings. The model incorporates architectural optimizations in the MLP structure to improve generalization, and its integration with Grad-CAM enables interpretability—a key novelty compared to black-box deep models.

We report per-class performance metrics to instill confidence that class imbalance handling is effective, and, as shown in the table, we see consistent improvement in minority classes. Although minority attack types pose challenges in rare scenarios, the application of class weighting combined with controlled resampling provided more balanced predictions amongst classes and less bias in predicting majority classes.

Experimental results demonstrate that CyberDetect-MLP outperforms existing baseline models in terms of accuracy, scalability, and explainability. The model achieved an accuracy of 98.87%, indicating robust detection capability even in noisy and imbalanced conditions. Performance comparisons, ablation studies, and visualization-supported analysis further confirm the effectiveness of the proposed methodology. By addressing limitations of scalability, explainability, and real-world applicability found in state-of-the-art solutions, this research contributes a practically viable and theoretically sound framework for IoT-centric security analytics. The implications of this work extend to secure smart city deployments, industrial IoT monitoring, and adaptive threat response systems, where both high performance and interpretability are crucial. The discussion of study-specific limitations is presented separately in Sect. 5.1 to maintain focus on the strengths and outcomes of the current research.

### Limitations of the study

Despite the strong performance of CyberDetect-MLP, the study has certain limitations. First, although the model is trained on the full TON_IoT dataset, it has not been validated on cross-domain datasets, such as CIC-IDS2017 or UNSW-NB15, which may limit its generalizability. Second, the current implementation uses offline evaluation; real-time performance under continuous streaming conditions is yet to be assessed. Third, although Grad-CAM provides interpretability, it is primarily designed for convolutional architectures and may offer limited insights for MLP-based decisions. These limitations open opportunities for further refinement and extension in future work.

## Conclusion and future work

We then proposed CyberDetect-MLP, a fast, scalable, and interpretable IDS framework fit for the resource-constrained nature of IoT. Courtesy of big data pipelines (Kafka/Flume-HDFS-Spark) and an advanced MLP design, it overcomes the main downsides of current IDS approaches, including poor scalability and transparency, and ineffective mitigation of high-dimensional IoT traffic. With respect to accuracy, recall, and F1-score, results on the TON_IoT dataset show that our approach outperforms several standard baselines. Model interpretability, as demonstrated by Grad-CAM and SHAP, improved trust in the model among administrators and in their response to incidents. Although this research was based on one specific dataset, our work can be further extended to include a cross-dataset cross-validation suite, as well as mitigation strategies for class imbalance and concept drift benchmarking. Quantitative evaluations of real-time inference latency and scalability over edge–fog deployments are also slated. This framework, demonstrated to be practical and reproducible, provides an IDS solution in a tool framework for securing IoT ecosystems, paving the way for future work that integrates explainable and scalable AI into next-generation cyber defense systems.

Looking ahead, several extensions can further improve the proposed system. First, validating the model across diverse datasets such as CIC-IDS2017 and UNSW-NB15 will test its cross-domain generalizability. Second, deploying the system in a real-time streaming environment using tools like Apache Kafka will evaluate its operational feasibility under live traffic. Third, integrating additional XAI methods suited for non-convolutional models may improve interpretability and decision transparency. Furthermore, adapting the framework to edge-computing scenarios or federated learning environments could ensure privacy-preserving and resource-efficient deployments. These directions offer rich potential for making CyberDetect-MLP a practical tool in next-generation IoT cybersecurity solutions.

## Data Availability

Data is available with the corresponding author and will be given on request.

## Materials availability

Materials used in this research are available with corresponding author and given on request.

## Code availability

The complete implementation of the proposed CyberDetect-MLP framework, including data preprocessing, model training, and evaluation scripts, is publicly available at: https://github.com/upender0123/CyberDetect-MLP.
